# Combined Focused Next-Generation Sequencing Assays to Guide Precision Oncology in Solid Tumors: A Retrospective Analysis from an Institutional Molecular Tumor Board

**DOI:** 10.3390/cancers14184430

**Published:** 2022-09-12

**Authors:** Thomas S. Tarawneh, Fiona R. Rodepeter, Julia Teply-Szymanski, Petra Ross, Vera Koch, Clemens Thölken, Jonas A. Schäfer, Niklas Gremke, Hildegard I. D. Mack, Judith Gold, Jorge Riera-Knorrenschild, Christian Wilhelm, Anja Rinke, Martin Middeke, Andreas Klemmer, Marcel Romey, Akira Hattesohl, Moritz Jesinghaus, Christian Görg, Jens Figiel, Ho-Ryun Chung, Thomas Wündisch, Andreas Neubauer, Carsten Denkert, Elisabeth K. M. Mack

**Affiliations:** 1 Department of Hematology, Oncology and Immunology, Philipps-University Marburg, Baldingerstraße, 35043 Marburg, Germany; 2 Institute of Pathology, Philipps-University Marburg, Baldingerstraße, 35043 Marburg, Germany; 3 Institute of Medical Bioinformatics and Biostatistics, Philipps-University Marburg, Hans-Meerwein-Straße 6, 35032 Marburg, Germany; 4 Department of Gynecology, Gynecologic Endocrinology and Oncology, Philipps-University Marburg, Baldingerstraße, 35043 Marburg, Germany; 5 Department of Gastroenterology and Endocrinology, Philipps-University Marburg, Baldingerstraße, 35043 Marburg, Germany; 6 Comprehensive Cancer Center Marburg, Philipps-University Marburg, Baldingerstraße, 35043 Marburg, Germany; 7 Department of Pulmonary and Critical Care Medicine, Philipps-University Marburg, Baldingerstraße, 35043 Marburg, Germany; 8 Department of Diagnostic and Interventional Radiology, Philipps-University Marburg, Baldingerstraße, 35043 Marburg, Germany

**Keywords:** molecular tumor board, next-generation sequencing, precision oncology, solid tumors, gene panels, ultra-low-coverage whole-genome sequencing

## Abstract

**Simple Summary:**

The molecular characterization of tumor tissues has become essential to classify tumors, assess prognoses and optimize treatment. However, there is still no consensus about the use of molecular diagnostics broadly across tumor types due to costs and limited evidence for the actual benefit of tumor-agnostic precision oncology. At our institution, we implemented three complementary NGS assays that are compatible with benchtop sequencing instruments as a diagnostic tool for identifying therapeutic targets and developing tailored treatment recommendations in a Molecular Tumor Board. Specifically, we used a 67-gene panel for the detection of short-sequence variants and copy-number alterations, a 53- or 137-gene panel for the detection of fusion transcripts and ultra-low-coverage whole-genome sequencing for detection of additional copy-number alterations outside the panel’s target regions. The Molecular Tumor Board was able to suggest personalized treatments to 75% of the patients, indicating that a combination of focused genetic diagnostics is highly informative for routine cancer care.

**Abstract:**

Background: Increasing knowledge of cancer biology and an expanding spectrum of molecularly targeted therapies provide the basis for precision oncology. Despite extensive gene diagnostics, previous reports indicate that less than 10% of patients benefit from this concept. Methods: We retrospectively analyzed all patients referred to our center’s Molecular Tumor Board (MTB) from 2018 to 2021. Molecular testing by next-generation sequencing (NGS) included a 67-gene panel for the detection of short-sequence variants and copy-number alterations, a 53- or 137-gene fusion panel and an ultra-low-coverage whole-genome sequencing for the detection of additional copy-number alterations outside the panel’s target regions. Immunohistochemistry for microsatellite instability and PD-L1 expression complemented NGS. Results: A total of 109 patients were referred to the MTB. In all, 78 patients received therapeutic proposals (70 based on NGS) and 33 were treated accordingly. Evaluable patients treated with MTB-recommended therapy (*n* = 30) had significantly longer progression-free survival than patients treated with other therapies (*n* = 17) (4.3 vs. 1.9 months, *p* = 0.0094). Seven patients treated with off-label regimens experienced major clinical benefits. Conclusion: The combined focused sequencing assays detected targetable alterations in the majority of patients. Patient benefits appeared to lie in the same range as with large-scale sequencing approaches.

## 1. Introduction

Precision oncology has the aim to use knowledge of tumor-specific molecular features to guide treatment decisions and improve patients’ outcomes [1]. Fundamental insights into cancer biology and the genetic basis of cancer; advances in molecular analysis techniques—most importantly, next-generation sequencing (NGS) technologies, and the availability of new molecularly targeted therapies (MTTs) have dramatically changed diagnostic and clinical approaches to malignant diseases. For example, gene-expression profiling platforms enable risk stratification and guide adjuvant treatment in luminal breast cancer [2,3] or allow to predict prognosis in diffuse-large B-cell lymphoma (DLBCL) [4]. Molecular characterization is also key to direct therapy in breast and colorectal cancer (CRC), for which anti-ERBB2 and anti-EGFR agents, respectively, represent the backbone of therapeutic regimens in certain subtypes defined by distinct genetic features such as *ERBB2* amplification or the absence of a *KRAS* mutation [5,6]. Moreover, oncogene addictive alterations involving, for example, *ALK*, *EGFR*, *BRAF* and *ABL1* genes in non-small cell lung cancer (NSCLC), melanoma and Chronic Myeloid Leukemia (CML) represent indications for MTTs instead of chemotherapy-based regimens as first-line treatment, thus enabling significantly prolonged survival, as well as long-lasting disease control, even in metastatic patients with tumors harboring these alterations [7,8,9,10,11]. Importantly, MTTs are generally well tolerated and have favorable safety profiles [12].

Even though the superiority of MTTs over conventional therapies is well documented within most approved indications, the concept of tissue-agnostic precision oncology and its utility across tumor types remains to be fully elucidated [13,14]. In particular, even with extensive NGS covering > 200 genes or the complete exome, less than 10% of patients experienced some kind of clinical benefit with MTTs according to recent reports [13,15,16,17,18,19,20,21]. The limited efficacy of precision oncology [22,23] is obviously contradictory to the expenditures for broad molecular profiling, as reported prices for NGS diagnostics range from EUR 500 to more than EUR 20,000 depending on the panel used [24,25,26,27]. Additionally, the examination, evaluation and interpretation of genomic data require expertise and a consistent interdisciplinary effort from bioinformaticians, molecular biologists and physicians and need to be performed in a timely manner in a clinical setting [28].

To date, *NTRK* fusions, tumor mutational burden (TMB) and microsatellite instability (MSI-high) are the only recognized tumor-agnostic biomarkers for which drugs are approved by the European Medicines Agency (EMA) and/or the US Food and Drug Administration irrespective of histology [29,30,31,32,33]. Therefore, the use of other biomarkers in an agnostic fashion for therapeutic purposes can be pursued only in clinical trials or as personalized off-label treatment.

We initiated a precision oncology program in 2018 at our institution. To provide a comprehensive clinically relevant genomic portrait of patients’ tumors, we used three complementary NGS assays. Specifically, we applied two commercially available panels, one to detect short-sequence variants (SSVs) (single nucleotide variants (SNVs) and insertion-deletions (INDELs)) and copy-number alterations (CNAs) (VariantPlex^®^, VP) and one to identify fusion transcripts (FT) (FusionPlex^®^, FP). Additionally, we adapted an ultra-low-coverage whole-genome sequencing (ulcWGS, <0.5× coverage) method developed in our laboratory as a module for the genetic characterization of Acute Myeloid Leukemia [34] to estimate numerical karyotypes and detect potentially actionable CNAs in regions not targeted by the VP panel. NGS was supplemented by immunohistochemistry (IHC) staining for programmed death-ligand 1 (PD-L1) and mismatch repair deficiency (MMRD)/microsatellite instability (MSI). Thus, our diagnostic core package focused on well-characterized and actionable molecular alterations, mostly targetable with drugs approved by EMA, while previously published precision oncology programs established diagnostic workflows based on larger gene panels, including up to 1700 genes or whole-exome sequencing (WES) [16,17,19,35,36,37]. The results and potential therapeutic options were discussed in the Molecular Tumor Board (MTB) by a panel of experts, including medical oncologists, pathologists, molecular biologists and other physicians from various specialties.

In this retrospective analysis, we examined how and to what extent a limited gene-sequencing approach that does not exploit the full capacities of currently available sequencing technologies provides clinically relevant information to guide individualized treatments in cancer patients.

## 2. Materials and Methods

### 2.1. Patients

A total of 109 patients with advanced-stage malignancies who had relapsed after initial remission, had progressed under standard of care (SoC) regimens or had rare disease entities without well-defined effective standard treatments were referred to our MTB between August 2018 and December 2021 by recommendation of the interdisciplinary or an organ-specific tumor board. There were no exclusion criteria based on patient age, tumor entity, known gene alterations (e.g., *KRAS* mutations) or patient performance status at the time of referral.

### 2.2. Tissue Samples and DNA Extraction

Tumor cells were enriched from formalin-fixed paraffin-embedded (FFPE) tissue slides from biopsy or surgical specimens by microdissection. For the VP and ulcWGS assays, DNA was extracted with the Maxwell RSC 48 (Promega, Walldorf, Germany), using the Maxwell RSC FFPE Plus DNA Kit or the blackPREP FFPE DNA Kit (Analytik Jena, Jena, Germany), respectively. RNA for the FP assay was isolated by applying the innuPREP FFPE total RNA Kit (Analytik Jena). Nucleic acids were quantified by using a Quantus Fluorometer with the QuantiFluor ONE dsDNA System (Promega) or a Qubit device (Thermo Fisher Scientific, Waltham, MA, USA) with dsDNA HS or RNA HS assays, as appropriate.

### 2.3. Library Preparation and Next Generation Sequencing

Libraries for targeted SSV and CNA detection were prepared from DNA, using the VariantPlex^®^ Solid Tumor panel (67 genes, Supplementary Table S1) (ArcherDX/Invitae, Boulder, CO, USA) according to the manufacturer’s instructions. For FT detection, sequencing libraries were constructed from RNA with the FusionPlex^®^ Solid Tumor panel (53 genes, Supplementary Table S2) and later (since October 2021) the FusionPlex^®^ Pan Solid Tumor panel (137 genes, Supplementary Table S3) (ArcherDX/Invitae), as outlined in the manufacturer’s protocol. Whole-genome libraries for genome-wide CNA analysis and numerical karyotype estimation were generated also from DNA, using the NEBNext Ultra II Kit (New England Biolabs, Ipswich, MA, USA), as described previously [34]. Sequencing was performed on Illumina MiSeq and MiniSeq Instruments (Illumina, San Diego, CA, USA), with read lengths of 2 × 150 bp.

### 2.4. Analysis of Genetic Variants

Raw reads from VP and FP assays were analyzed by using the Archer Analysis 6.1 and 6.2 pipeline (ArcherDX/Invitae) from a cloud-based client (Archer unlimited) or a local server, using default settings. For variant calling, alterations reported by the software were filtered for a coverage of over 100×, a variant allele frequency (VAF) of 5% and a gnomAD [38] frequency below 5%. Karyotyping based on reads obtained from ulcWGS was performed by using a proprietary tool that involves a read-depth approach. In this study, we applied a modified version of our original Python-based CAI[N] algorithm [34] that was implemented in the R programming environment (CORIANDR, ChrOmosomal abeRration Identifier AND Reporter in R). A Panel of Normals (PON) constructed from DNA isolated from total leukocytes of 20 healthy donors (10 males and 10 females) was used as a reference for karyotype calculations (more details about CORIANDR are given in Supplementary Materials). Two researchers independently evaluated the initial CORIANDR outputs to exclude potential artifacts and determine numerical karyotypes. Disagreements were resolved by consensus after joint inspection and discussion of the results.

### 2.5. Variants Interpretation and Therapy Recommendations

All variants (SSVs, CNAs and FTs) that passed standard filters of the analysis tools were reviewed for potential artifacts, and all non-artificial variants were assessed for clinical relevance based on the current literature and publicly available databases (OncoKB [39], CIViC [40], JAX CKB [41], ClinVar [42,43] and gnomAD [38]). The Variant Interpretation for Cancer Consortium Standard Operating Procedure (VICC-SOP) [44] has been formally applied for classifying pathogenicity since October 2021. Evidence supporting therapeutic approaches was rated by using the European Society of Medical (ESMO) Scale for Clinical Actionability of molecular Targets (ESCAT) [45] and the German Cancer Consortium (DKTK) [46] scales. Study options were assessed by using the clinicaltrials.gov-database (https://clinicaltrials.gov/ (accessed on 1 August 2022)), but due to the COVID-19 pandemic, the search was limited to studies recruiting in Germany since March 2020. Therapy recommendations were prioritized according to the level of supporting evidence and assumed feasibility of clinical implementation. Phase-I trials were generally not included in MTB recommendations for patients requiring timely initiation of treatment.

### 2.6. Immunohistochemistry for PD-L1 and MMRD/MSI

PD-L1 immunohistochemistry on FFPE slices was performed by using anti-PD-L1 antibody clone E1L3N (1:1000, Cell Signaling, Frankfurt am Main, Germany) on the BondMax™ staining device (Leica Biosystems GmbH, Wetzlar, Germany). Staining intensity and distribution were evaluated on tumor cells (TPS-score), immune cells (IC-score) and as a combined score (CPS-score) [47].

MMRD/MSI testing was performed by immunohistochemical staining for mismatch repair proteins MLH1, PMS2, MSH2 and MSH6 (antibodies 1:50, Cell Marque/Sigma Aldrich, Taufkirchen, Germany), with loss of nuclear staining for one or more proteins indicating a mismatch repair deficiency of the tumor.

### 2.7. Outcome Assessment

Measures of the outcome included the progression-free survival (PFS), PFS ratio (PFSr) (time to progression from matched or unmatched therapy initiation (PFS2) divided by time to progression associated with the last prior systemic therapy (PFS1)) [48], objective response rate (ORR) and patient reported outcomes (PROs) when available.

We considered a 3-month and a 6-month time to progression cutoff to define minor benefit or major benefit, respectively, with matched treatment [17,48,49,50]. Minor benefit was also recorded for patients with less than a 3-month follow-up after therapy initiation who showed clinical response to treatment (e.g., improvement of symptoms and decrease of tumor marker).

Patients who progressed or died while on treatment before the 3-month cutoff or permanently stopped treatment due to toxicity or intolerability were considered progressors/non responders.

### 2.8. Statistical Analyses

A comparison of PFS in patients treated with MTB-recommended therapies and non-recommended therapies was performed by Kaplan–Meier analysis in GraphPad Prism software, version 8.1.2 (GraphPad Software Inc., San Diego, CA, USA). PFS ratios were compared by using a Mann–Whitney test, using Jamovi Software, version 2.3.2 (The jamovi project).

## 3. Results

### 3.1. Clinical Implementation of Precision Oncology—Outline

**Figure 1:**
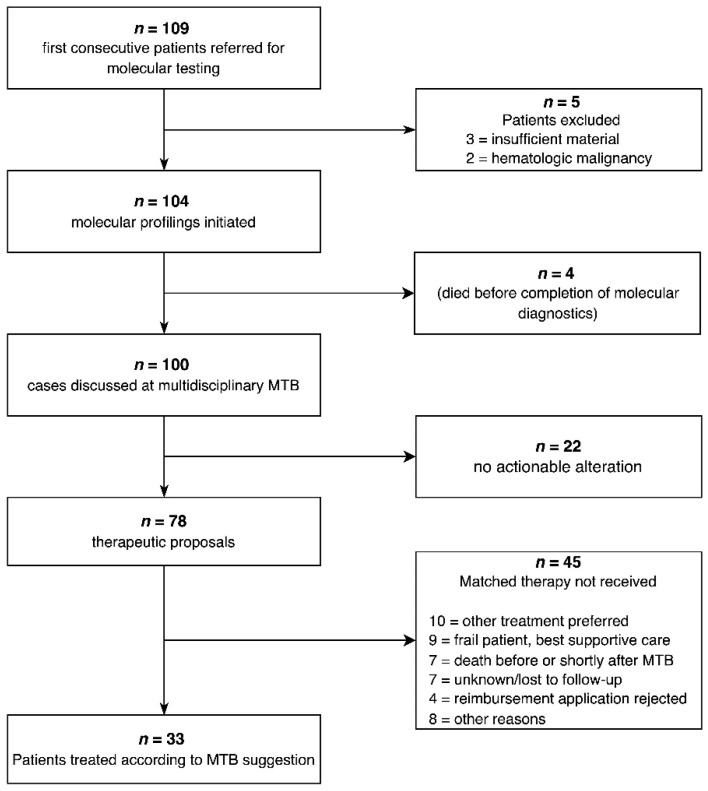
CONSORT diagram—outline of the implementation of precision oncology in clinical practice. Initially, 109 patients were referred for molecular testing, aiming at individual targeted therapies. Thirty-three patients (30%) received matched therapies.

From August 2018 to December 2021, 109 patients were referred to our MTB for molecular testing and evaluation of molecular-stratified therapies. Three patients were excluded from our analyses because of insufficient tissue samples to carry out the diagnostics. Two patients with hematologic malignancies were also excluded from this analysis, as the precision oncology approach was requested preferentially for patients with solid tumors, so that we decided to focus our retrospective analysis on these entities. Additionally, four patients died before the molecular diagnostics was concluded. Finally, 100 patients were discussed in the MTB, with 78 obtaining a therapeutic suggestion, while the remaining 22 had no actionable target. Thirty-three patients received molecularly matched treatment. A total of 16 of the 45 patients not receiving molecularly matched therapy (approximately 36%) did not receive any further treatment because of death or clinical deterioration. The remaining patients did not receive molecularly matched therapy because of physician’s preference for other regimens (10/45), rejection of reimbursement applications by health insurance companies (4/45) or other reasons (8/45). In all, 7/45 patients with an MTB-therapeutic suggestion were lost to follow-up (Figure 1).

### 3.2. Patients Characteristics

Our retrospective analysis included 104 patients with solid malignancies who were referred to our MTB for molecular testing to identify matched targeted treatment, which was performed successfully. In all, 50% of the patients were females. The median age at the time of referral was 57.5 years. Most patients (100; 96.1%) had metastatic disease; however, 41 patients had been previously treated with a curative intent and had only subsequently relapsed or progressed to metastatic stage. The most common tumors were lower gastrointestinal (GI) tract tumors (18/104; 17.3%), followed by neuroendocrine neoplasms (13/104; 12.5%) and sarcomas (12/104; 11.5%). Over 50% of patients had received three or more lines of treatment (prior to MTB or to initiation of molecularly matched therapy; Table 1).

### 3.3. Molecular Testing and Therapeutic Suggestions

**Figure 2:**
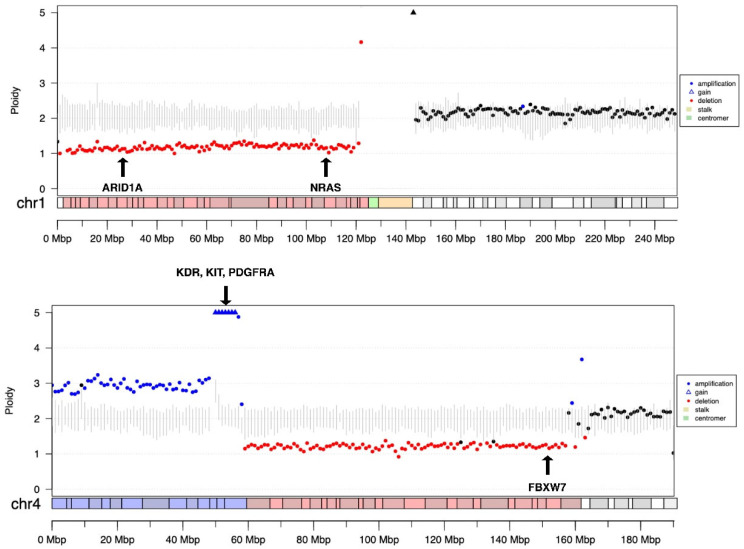
CNAs detected via ulcWGS. Chromosome (Chr.) plots generated by CORIANDR for Chrs. 1 and 4 of a patient with sarcoma. Amplifications of KDR, KIT and PDGFRA were confirmed by ulcWGS on chromosome 4 (add(q)12), as reported by VP analyses. Additionally, the ulcWGS detected a heterozygous deletion of chromosome 1p (including the region of the ARID1A gene, not target by VP, and NRAS gene, heterozygous deletion also detected by VP) and a partial loss of Chr. 4q (a partial loss of FBXW7 gene with Copy Number (CN) of 0.62 was also reported by VP assay). Other low-level CN gains and losses detected via VP and ulcWGS are displayed in Supplementary Materials Figure S1.

**Figure 3:**
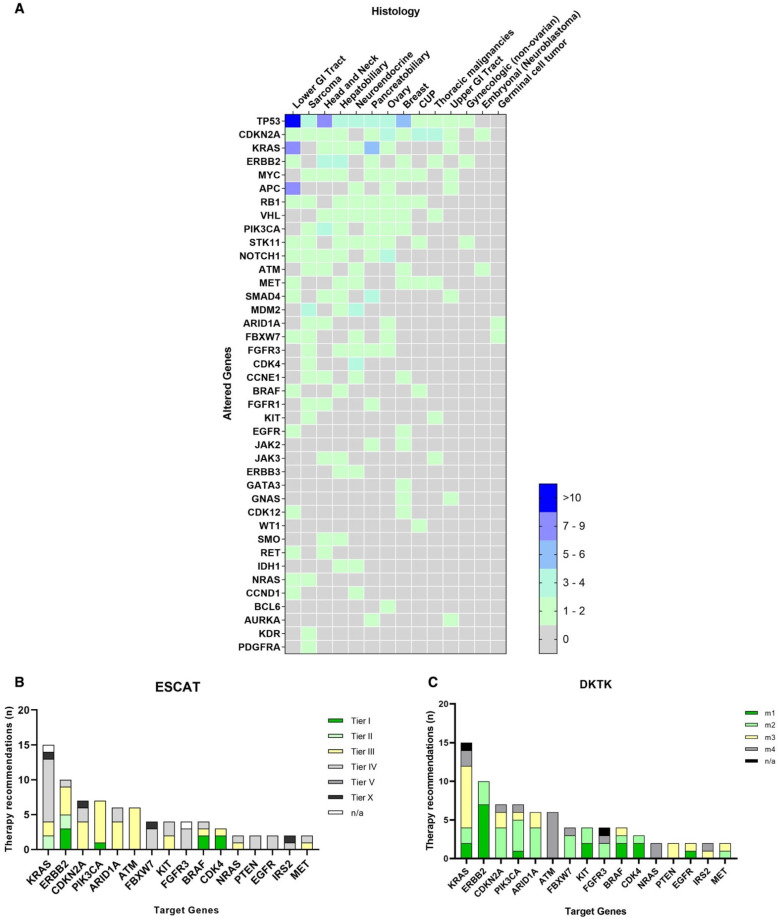
SSVs and CNAs in *n* = 104 tumors identified by combined focused sequencing assays. (**A**) List of altered genes divided by histology and according to incidence in our cohort. Only alterations observed in at least two samples are depicted. (**B**,**C**) Most common actionable gene alterations classified according to ESCAT (**B**) and DKTK (**C**) scales of actionability of genetic alterations based on supporting evidence. Genes reported in panels B and C provided a therapeutic rationale in at least two cases. Cf. also Figure S2 and Table S5 in the supplementary materials.

Complete NGS diagnostic (FP + ulcWGS + VP) testing was performed in 91 patients. Due to insufficient tissue samples, one panel or a composite of two panels was performed in the remaining 13 patients: VP and FP were performed in six patients, ulcWGS and VP in five and only VP in two patients. SSVs were detected in 69 patients, whereas CNAs were detected in 64 patients. A total of 95 CNAs were found through VP, 55 of which (58%) were confirmed via ulcWGS. We also detected 76 additional CNAs via ulcWGS, 38/76 (50%) in regions not targeted by the VP panel (Figure 2). The most commonly altered genes were *TP53*, *CDKN2A*, *KRAS*, *ERBB2*, *MYC* and *APC* (Figure 3A). The levels of evidence for therapeutic actionability of the identified alterations were mainly Tier III or m2, respectively, according to the ESCAT and DKTK scales [45,46] (Figure 3B,C). Six patients had FTs (Table 2).

A total of 108 therapeutic suggestions were made for 78 patients. Particularly, 50 patients received one therapeutic suggestion, and 28 received two or more. Moreover, 70 patients received suggestions based on NGS results. For two patients with actionable alterations (*FBXW7* deletion and pathogenic *IDH1* mutation), the MTB prioritized other treatment options because of the availability of a tumor-entity-specific clinical trial and low VAF, respectively. Furthermore, NGS enabled the diagnosis of primary tumor entity in three patients who had been initially diagnosed with cancer of unknown primary (CUP) (Table 2). For two of these three patients (prostate carcinoma and *ALK*-fusion-positive DLBCL), the MTB suggested SoC treatment. In the prostate cancer case, the decision was made due to the lack of actionable alterations. In the *ALK*-fusion-positive DLBCL case, SoC therapy was suggested because the patient had not received a systemic therapy appropriate for the disease entity yet (Table 3). For the third patient (gastric cancer), the board suggested a combination of an immune checkpoint inhibitor with a multi-tyrosine-kinase inhibitor (off-label) because of hematologic toxicities from prior chemotherapy and the impossibility to include the patient in a clinical trial with a targeted agent for CLDN18.2 (Table 2). Three patients received an off-label therapy suggestion based on data other than NGS results (PD-L1 positivity in IHC, MSI-high and data from a clinical trial in the same tumor entity, respectively).

### 3.4. Outcomes with MTB-Recommended Therapy

**Figure 4:**
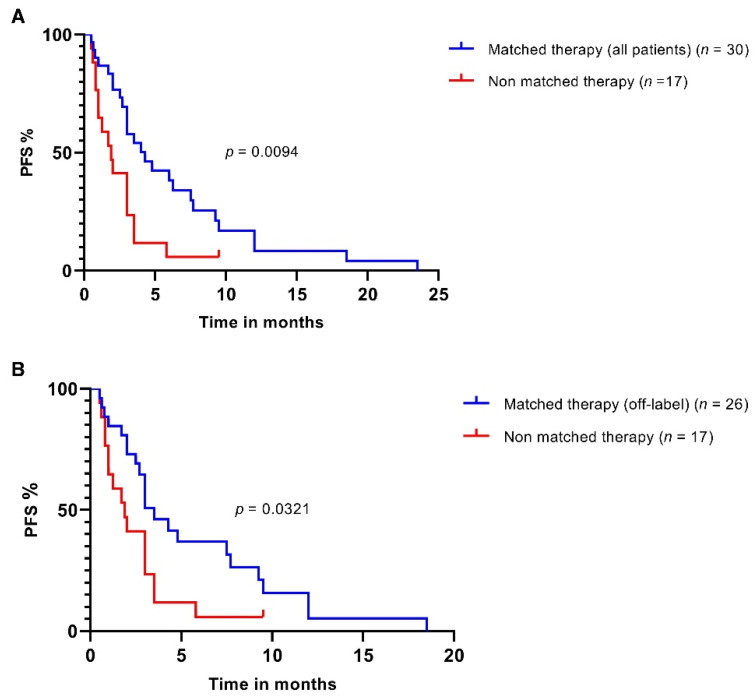
Outcome of patients after molecular profiling and discussion of cases by the MTB. (**A**) PFS of patients treated according to MTB recommendation (all patients, *n* = 30) compared to patients receiving other systemic treatment (*n* = 17). (**B**) PFS of patients treated by off-label regimens according to MTB recommendation (*n* = 26) vs. patients receiving other systemic treatment (*n* = 17). *p*-values were calculated with a log-rank test.

Of 100 patients discussed at the MTB, 33 received molecularly matched therapy. At the time this manuscript was written, 2/33 were not evaluable due to short follow-up, and 1/33 was lost to follow-up. Of the remaining 67 patients who did not receive molecularly matched therapy, 14/67 did not receive any further systemic treatment, 14/67 were lost to follow-up, 11/67 died shortly after or before MTB, 8/67 were still on previous line treatment and 3/67 were not evaluable due to short follow-up. Ultimately, 47/100 patients received systemic therapy and were evaluable for outcome. We compared the PFS of patients receiving MTB-recommended therapy to patients who were treated with other systemic therapies according to physician’s choice by Kaplan–Meier analysis. The PFS in patients treated with matched therapy (recommended by the MTB) (*n* = 30) was prolonged significantly according to the log-rank test (4.3 vs. 1.9 months, *p* = 0.0094, Figure 4A). The PFS in the subgroup of patients treated according to MTB suggestion with off-label regimens (*n* = 26) was 3.5 months, which was also significantly longer than in those treated with other therapies (*n* = 17) (Figure 4B).

The PFSr was evaluable in 38 patients receiving systemic treatment after the MTB. The median PFSr in the subgroup receiving therapy suggested by the MTB was 1.71 in the all-patients group (*n* = 23) and 1.33 in the subgroup receiving off-label therapies (*n* = 19). The PFSr in patients receiving other therapies (*n* = 15) was 0.61. Despite the numerical difference, this did not reach statistical significance (Mann–Whitney U test, *p* = 0.052 and *p* = 0.083, respectively, for the all-patients population and for patients receiving off-label treatments).

According to our initially established parameters, of 30 evaluable patients receiving MTB recommended treatment, 9 (30%) achieved no benefit, 11 (36.7%) achieved a minor benefit and 10 (33.3%) achieved a major benefit (Table 3). Thus, of the 104 patients who underwent molecular profiling of their tumor, 9.6% experienced a highly relevant clinical improvement (major clinical benefit) through precision oncology.

## 4. Discussion

Recommending a therapy in advanced-stage cancer patients can be extremely challenging, especially when SoC treatments are no longer a viable option. Despite the use of a focused approach to gene diagnostics that did not require high-throughput sequencing equipment, our MTB was able to suggest further rational treatments in about 75% of patients. The rates of implementation of the suggested therapies (approximately 32%) and patient benefits (approximately 10%) lay within the same ranges of previously published reports of other precision-oncology programs [13,17,18,19,20,21].

Although patients were preferentially referred to our multidisciplinary MTB to identify potentially actionable alterations, NGS uncovered the specific tumor entity in three out of eight patients with CUP. This enabled clinicians to choose an appropriate treatment regardless of additional alterations. One patient achieved a complete remission with SoC chemotherapy matched to the tumor type, which was still ongoing at the time this manuscript was written. Thus, in line with other reports [17,51], our experience confirms that gene sequencing can guide treatment and therefore improve prognosis in patients with CUP. Interestingly, in all three cases, histologic classification was possible based on the detection of characteristic FTs through FP analysis. This supports the importance of carrying out both DNA and RNA sequencing if no system-wide approach is used. Indeed, while DNA assays are fundamental for SSV detection, fusions are more effectively characterized by RNA-based assays [52,53]. Furthermore, the addition of ulcWGS and IHC staining for MMRD/MSI and PD-L1 to the two commercially available panels allowed us to investigate the presence of further potentially targetable CNAs, as well as therapeutic rationales for the use of immune checkpoint inhibitors. Moreover, ulcWGS can, in principle, add valuable information not only to small, targeted panels, as used here, but also to larger ones, as the assay represents the only unbiased component of our sequencing strategy and requires only minimal sequencing resources. On the other hand, calculated numerical karyotyping opens up the perspective to include “cytogenetic” information in the molecular characterization of solid tumors [54] on a routine basis, which might be used to develop genetic-risk stratification systems analogous to hematologic malignancies such as acute myeloid leukemia [55,56].

Based on the current literature, only a small fraction of patients (<10% of sequenced cases) achieve clinical benefits from precision oncology [13,17,18,19,20,21]. This small percentage appears not to change meaningfully with different sequencing techniques or by expanding the number of genes analyzed. Importantly, the PERMED-01 trial revealed that the use of WES provided no more clinically relevant information than gene panels analyzing >300 to around 800 genes [36]. Additionally, a recent study demonstrated that, in NSCLC, smaller panels are more sensitive than larger ones in detecting alterations in commonly altered genes (e.g., *EGFR* and *BRAF*) and therefore represent a more useful tool at disease diagnosis [57].

Our retrospective study confirmed that a small subgroup of highly pretreated advanced-stage cancer patients can benefit from precision oncology. Most importantly, we could identify this subgroup with smaller gene panels. This is remarkable because, while NGS costs have dramatically decreased in recent years, especially in terms of prices of reagents and personnel time directly involved into sequencing [24], expanding gene analyses to bigger gene panels or even WES increases the time necessary for data analysis and interpretation. First, larger outputs need more sophisticated bioinformatical processing to exclude artifacts and benign variants. Second, data interpretation and classification of detected variants can be very time-consuming, also due to the emergence of variants of uncertain significance.

Although the diagnostic approach outlined here allowed us to identify actionable alterations in the majority of patients, most alterations were targetable through drugs not approved for patients’ specific disease; that is, access to treatment was only possible through clinical trials, off-label therapies or compassionate use programs. However, although trial options were evaluated for all patients prior to the MTB, early clinical studies were only recommended in exceptional cases, as these options were regarded as being non-feasible due to the limited availability of treatment slots. Moreover, due to travel restrictions issued by many countries in 2019/2020 following the COVID-19 pandemic, the search for clinical trials was generally limited to trials recruiting in Germany. The MTB therefore preferentially recommended off-label therapies, for which reimbursement by the patients’ health insurance had to be formally requested. However, evidence underlying the MTB recommendation was often not considered sufficient to grant coverage. These observations highlight that the actual implementation of precision oncology was hampered more by limited access to drugs, rather than by the lack of potential targets identified by our focused molecular diagnostic workup. As underlined in a recent publication, an interdisciplinary approach combining diagnostic and medical interventions in future precision oncology programs might represent a possible solution to increase the rates of access to personalized therapies [58].

Potential interpretation biases of this study arise from the lack of randomization, its retrospective nature and the small patient population with a large heterogeneity in terms of diagnoses and previous treatments. Moreover, while we observed a significant prolongation of PFS in patients receiving molecularly matched therapy, 15% of patients not receiving matched therapy were lost to follow-up, as they continued their treatment at a different oncology clinic or practice, and their outcomes were therefore not evaluable.

Another relevant aspect to consider is the timing when to perform NGS in cancer patients. Although the presence of tumor-agnostic markers (*NTRK* fusions, TMB and MSI) provides the premise for the use of sequencing in all tumors [59], ESMO guidelines recommend NGS just for patients with non-squamous NSCLC, ovarian carcinoma, prostate carcinoma and cholangiocarcinoma, so that, other than in these indications, the use of NGS should be considered on a case-by-case basis [60]. However, data from our analysis also highlight that about 20% of patients with a therapeutic suggestion from our MTB did not receive any systemic treatment following the MTB because of death or poor performance status not allowing further therapy. Additionally, in some cases, insurance companies agreed to cover expenses for off-label treatment only conditionally after failure of several lines of therapy, a situation in which patients are often unlikely to draw clinical benefit. Our preliminary data therefore support the notion that NGS testing should be taken into consideration rather early in the course of a disease in order to avoid the waiving of possible matched therapies due to compromised patient performance status after several lines of therapy [61].

## 5. Conclusions

Combined focused sequencing assays to identify SSVs, CNAs and FTs in the most commonly altered and/or actionable genes in tumors, along with ulcWGS and IHC staining for PD-L1 and MMRD/MSI, represent a powerful tool to explore additional treatment options in cancer patients with otherwise limited or not well-defined further lines of therapy. This approach provides a reasonable first step for precision oncology and a good alternative to larger gene panels and WES, especially in centers where the implementation of these technologies is not (yet) feasible in the clinical practice.

## Figures and Tables

**Table 1:** Patient characteristics of 104 patients whose tumors were subjected to molecular testing for precision oncology.

	*N*	Percentage (%) or Range
**Total**	104	
**Sex**		
Female	52	(50)
Male	52	(50)
**Age**	57.5 (median)	22–77 (range)
**Stage at presentation**		
Metastatic disease	100	(96.1)
Localized	3	(2.9)
Locally advanced	1	(1.0)
**Initial therapeutic intent**		
Primary metastatic, palliative intent	63	(60.6)
Initially curative intent	41	(39.4)
**Tumor type**		
Lower GI tract	18	(17.3)
Neuroendocrine neoplasms	13	(12.5)
Sarcoma	12	(11.5)
Head and neck	11	(10.6)
Hepatobiliary	10	(9.6)
CUP	8	(7.7)
Pancreatobiliary	7	(6.7)
Breast	6	(5.8)
Ovary	6	(5.8)
Thoracic malignancies	5	(4.8)
Upper GI tract	3	(2.9)
Gynecologic (non-ovarian)	2	(1.9)
Germ cell tumor	2	(1.9)
Embryonal (Neuroblastoma)	1	(1.0)
**Previous lines of treatment**		
0	1	(1.0)
1	13	(12.5)
2	33	(31.7)
3	25	(24.0)
>3	32	(30.8)
	3 (median)	0–14 (range)

Lower-GI tract includes CRC, anal carcinoma and appendiceal carcinoma; neuroendocrine neoplasms includes neuroendocrine tumors and carcinomas; hepatobiliary includes hepatocellular carcinoma and cholangiocarcinoma and gallbladder carcinoma; pancreatobiliary includes pancreatic carcinoma and ampullary carcinoma; thoracic malignancies include NSCLC, small-cell lung cancer, thymic carcinoma and thymoma; germ cell tumors include yolk-sac tumor and testicular tumor; upper-GI tract includes duodenal carcinoma and adenocarcinoma of the gastroesophageal junction.

**Table 2:** Fusion transcripts and respective clinical relevance identified in our MTB cohort.

Diagnosis	Fusion	Clinical Relevance
CUP	TMPRSS2-ERG	Changed diagnosis from CUP to prostate carcinoma
CUP	SEC31A-ALK	Changed diagnosis from CUP to ALK-positive DLBCL
CUP	CLDN18-ARHGAP26	Changed diagnosis from CUP to gastric carcinoma
Pancreatic carcinoma	EML4-ALK (previously not characterized transcript—E2;A18) *	Molecular rationale for ALK-inhibitor (Alectinib)
Cholangiocarcinoma	FGFR2-ARHGAP24	Molecular rationale for FGFR-inhibitor (Pemigatinib)
Adenoid cystic Carcinoma	MYB-NFIB	Characteristic of adenoid cystic carcinoma, no evidence for direct actionability

* Additional information in Supplementary Figure S3.

**Table 3:** Patients with major clinical benefit following MTB recommended therapy.

Diagnosis	Therapeutic Rationale	MTB Recommendation	EL ^γ^ ESCAT	EL ^γ^ DKTK	Label	PFS2	PFS1	PFSr	Outcome
CUP	*TMPRSS2-ERG* fusion (changed diagnosis from CUP to prostate cancer)	Docetaxel + ADT	n/a	n/a	On	6	3.5	1.7	SD for 6 months
Acinic cell carcinoma	*ATM* del	Niraparib + Carboplatin	III-B	m4	Off	12	n/a	n/a	SD for 12 months
CUP	*SEC31A-ALK* fusion (changed diagnosis from CUP to CD20-negative *ALK* + DLBCL)	Chemotherapy (CHOEP)	n/a	n/a	On	23.5	10.5	2.2	CR, ongoing at almost-2-year follow-up
CRC	*BRAF* V600E	Encorafenib + Binimetinib + Cetuximab	I-A	m1a	Off	7.5	3	2.5	SD for 7.5 months
Sinonasal adenocarcinoma	*ARID1A* deletion	Pembrolizumab	III-A	m2b	Off	12	n/a	n/a	SD for 12 months
Steroid cell tumor	*RET* Y791F	Cabozantinib	IV-A	m3	Off	9.5	1	9.5	Decrease of Tumor marker and SD for 9.5 months
CUP	*BRAF* V600E	Encorafenib + Binimetinib	III-A	m2a	Off	18.5	3	6.2	PR and SD for 18 months
TNBC	*EGFR* amplification	Cetuximab + Capecitabine	IV-A	m1c	Off	9.25	2	4.6	SD for 9.25 months
Sarcoma	*KDR*, *KIT*, *PDGFRA* Amplification	Pazopanib	IV-A	m3	On	6.25	14	0.4	SD for 6.25 months
Pancreatic cancer	*EML4-ALK* Fusion	Alectinib monotherapy	III-B	m4	Off	7.7	1	7.7	PR and SD for 7.7 months

ADT, androgen-deprivation therapy; CHOEP, Cyclophosphamide, Doxorubicin, Vincristine, Etoposide and Prednisone; CR, complete remission; EL, evidence level; n/a, not applicable; PR, partial remission; SD, stable disease; TNBC, triple-negative breast cancer. ^γ^ Supplementary Table S4.

## Data Availability

Data presented in this research paper will be made available upon request to the corresponding authors.

## Supplementary Materials

The following supporting information can be downloaded at https://www.mdpi.com/article/10.3390/cancers14184430/s1. Tarawneh-et-al_Supplementary Materials.pdf. References [62,63,64,65,66,67,68,69,70,71,72,73,74,75,76,77,78,79] are cited in the Supplementary Materials. Figure S1: Chromosome (Chr.) plots generated by CORIANDR for Chr. 3, 8, 11, 12 of a patient with sarcoma; Figure S2: Distribution of genetic alterations across cancer types and therapeutic suggestions; Figure S3: A non-canonical EML4-ALK fusion in a patient with pancreatic cancer; Table S1: Target regions of the VariantPlex Solid Tumor Panel (ArcherDx/Invitae); Table S2: Target genes of the FusionPlex Solid Tumor Panel (ArcherDx/Invitae); Table S3: Target genes of the FusionPlex Pan Solid Tumor Panel (ArcherDx/Invitae); Table S4: Patients with major clinical benefit following MTB recommended therapy; Table S5: Genetic alterations in *n* = 104 patients referred to the MTB.

## References

[1] Rodriguez H., Zenklusen J.C., Staudt L.M., Doroshow J.H., Lowy D.R. (2021). The next horizon in precision oncology: Proteogenomics to inform cancer diagnosis and treatment. Cell.

[2] Sparano J.A., Gray R.J., Makower D.F., Pritchard K.I., Albain K.S., Hayes D.F., Geyer C.E., Dees E.C., Goetz M.P., Olson J.A. (2018). Adjuvant Chemotherapy Guided by a 21-Gene Expression Assay in Breast Cancer. N. Engl. J. Med..

[3] Vieira A., Schmitt F. (2018). An Update on Breast Cancer Multigene Prognostic Tests—Emergent Clinical Biomarkers. Front. Med..

[4] Merdan S., Subramanian K., Ayer T., Van Weyenbergh J., Chang A., Koff J.L., Flowers C. (2021). Gene expression profiling-based risk prediction and profiles of immune infiltration in diffuse large B-cell lymphoma. Blood Cancer J..

[5] Slamon D.J., Leyland-Jones B., Shak S., Fuchs H., Paton V., Bajamonde A., Fleming T., Eiermann W., Wolter J., Pegram M. (2001). Use of Chemotherapy plus a Monoclonal Antibody against HER2 for Metastatic Breast Cancer That Overexpresses HER2. N. Engl. J. Med..

[6] Bokemeyer C., Bondarenko I., Makhson A., Hartmann J.T., Aparicio J., de Braud F., Donea S., Ludwig H., Schuch G., Stroh C. (2009). Fluorouracil, Leucovorin, and Oxaliplatin with and Without Cetuximab in the First-Line Treatment of Metastatic Colorectal Cancer. J. Clin. Oncol..

[7] Solomon B.J., Mok T., Kim D.-W., Wu Y.-L., Nakagawa K., Mekhail T., Felip E., Cappuzzo F., Paolini J., Usari T. (2014). First-Line Crizotinib versus Chemotherapy in *ALK*-Positive Lung Cancer. N. Engl. J. Med..

[8] Mok T., Camidge D., Gadgeel S., Rosell R., Dziadziuszko R., Kim D.-W., Pérol M., Ou S.-H., Ahn J., Shaw A. (2020). Updated overall survival and final progression-free survival data for patients with treatment-naive advanced ALK-positive non-small-cell lung cancer in the ALEX study. Ann. Oncol..

[9] Mok T.S., Wu Y.L., Ahn M.J., Garassino M.C., Kim H.R., Ramalingam S.S., Shepherd F.A., He Y., Akamatsu H., Theelen W.S. (2017). Osimertinib or Platinum–Pemetrexed in EGFR T790M–Positive Lung Cancer. N. Engl. J. Med..

[10] Long G.V., Flaherty K.T., Stroyakovskiy D., Gogas H., Levchenko E., De Braud F., Larkin J., Garbe C., Jouary T., Hauschild A. (2017). Dabrafenib plus trametinib versus dabrafenib monotherapy in patients with metastatic BRAF V600E/ K-mutant melanoma: Long-term survival and safety analysis of a phase 3 study. Ann. Oncol..

[11] O’Brien S.G., Guilhot F., Larson R.A., Gathmann I., Baccarani M., Cervantes F., Cornelissen J.J., Fischer T., Hochhaus A., Hughes T. (2003). Imatinib Compared with Interferon and Low-Dose Cytarabine for Newly Diagnosed Chronic-Phase Chronic Myeloid Leukemia. N. Engl. J. Med..

[12] Schirrmacher V. (2019). From chemotherapy to biological therapy: A review of novel concepts to reduce the side effects of systemic cancer treatment (Review). Int. J. Oncol..

[13] Singh A.P., Shum E., Rajdev L., Cheng H., Goel S., Perez-Soler R., Halmos B. (2020). Impact and Diagnostic Gaps of Comprehensive Genomic Profiling in Real-World Clinical Practice. Cancers.

[14] Remon J., Dienstmann R. (2018). Precision oncology: Separating the wheat from the chaff. ESMO Open.

[15] Presley C.J., Tang D., Soulos P.R., Chiang A.C., Longtine J.A., Adelson K.B., Herbst R.S., Zhu W., Nussbaum N.C., Sorg R.A. (2018). Association of Broad-Based Genomic Sequencing with Survival Among Patients with Advanced Non–Small Cell Lung Cancer in the Community Oncology Setting. JAMA J. Am. Med. Assoc..

[16] Niogret J., Dalens L., Truntzer C., Chevrier S., Favier L., Lagrange A., Coudert B., Fraisse C., Foucher P., Zouak A. (2021). Does large NGS panel analysed using exome tumour sequencing improve the management of advanced non-small-cell lung cancers?. Lung Cancer.

[17] Cobain E.F., Wu Y.-M., Vats P., Chugh R., Worden F., Smith D.C., Schuetze S.M., Zalupski M.M., Sahai V., Alva A. (2021). Assessment of Clinical Benefit of Integrative Genomic Profiling in Advanced Solid Tumors. JAMA Oncol..

[18] Fernandes G.S., Marques D.F., Girardi D.M., Braghiroli M.I.F., Coudry R.A., Meireles S.I., Katz A., Hoff P.M. (2017). Next-generation sequencing-based genomic profiling: Fostering innovation in cancer care?. Clinics.

[19] Hoefflin R., Lazarou A., Hess M., Reiser M., Wehrle J., Metzger P., Frey A., Becker H., Aumann K., Berner K. (2021). Transitioning the Molecular Tumor Board from Proof of Concept to Clinical Routine: A German Single-Center Analysis. Cancers.

[20] Varnier R., Le Saux O., Chabaud S., Garin G., Sohier E., Wang Q., Paindavoine S., Pérol D., Baudet C., Attignon V. (2019). Actionable molecular alterations in advanced gynaecologic malignancies: Updated results from the ProfiLER programme. Eur. J. Cancer.

[21] Massard C., Michiels S., Ferté C., Le Deley M.-C., Lacroix L., Hollebecque A., Verlingue L., Ileana E., Rosellini S., Ammari S. (2017). High-Throughput Genomics and Clinical Outcome in Hard-to-Treat Advanced Cancers: Results of the MOSCATO 01 Trial. Cancer Discov..

[22] Marquart J., Chen E.Y., Prasad V. (2018). Estimation of the Percentage of US Patients with Cancer Who Benefit from Genome-Driven Oncology. JAMA Oncol..

[23] Prasad V. (2016). Perspective: The precision-oncology illusion. Nature.

[24] Pruneri G., De Braud F., Sapino A., Aglietta M., Vecchione A., Giusti R., Marchiò C., Scarpino S., Baggi A., Bonetti G. (2021). Next-Generation Sequencing in Clinical Practice: Is It a Cost-Saving Alternative to a Single-Gene Testing Approach?. PharmacoEconomics—Open.

[25] Schwarze K., Buchanan J., Fermont J.M., Dreau H., Tilley M.W., Taylor J.M., Antoniou P., Knight S.J.L., Camps C., Pentony M.M. (2019). The complete costs of genome sequencing: A microcosting study in cancer and rare diseases from a single center in the United Kingdom. Genet. Med..

[26] Christensen K.D., Phillips K.A., Green R.C., Dukhovny D. (2018). Cost Analyses of Genomic Sequencing: Lessons Learned from the MedSeq Project. Value Health.

[27] Schwarze K., Buchanan J., Taylor J.C., Wordsworth S. (2018). Are whole-exome and whole-genome sequencing approaches cost-effective? A systematic review of the literature. Genet. Med..

[28] Damodaran S., Berger M.F., Roychowdhury S. (2015). Clinical Tumor Sequencing: Opportunities and Challenges for Precision Cancer Medicine. American Society of Clinical Oncology Educational Book.

[29] Hong D.S., DuBois S.G., Kummar S., Farago A.F., Albert C.M., Rohrberg K.S., van Tilburg C.M., Nagasubramanian R., Berlin J.D., Federman N. (2020). Larotrectinib in patients with TRK fusion-positive solid tumours: A pooled analysis of three phase 1/2 clinical trials. Lancet Oncol..

[30] Doebele R.C., Drilon A., Paz-Ares L., Siena S., Shaw A.T., Farago A.F., Blakely C.M., Seto T., Cho B.C., Tosi D. (2020). Entrectinib in patients with advanced or metastatic NTRK fusion-positive solid tumours: Integrated analysis of three phase 1–2 trials. Lancet Oncol..

[31] Marabelle A., Fakih M., Lopez J., Shah M., Shapira-Frommer R., Nakagawa K., Chung H.C., Kindler H.L., Lopez-Martin J.A., Miller W.H. (2020). Association of tumour mutational burden with outcomes in patients with advanced solid tumours treated with pembrolizumab: Prospective biomarker analysis of the multicohort, open-label, phase 2 KEYNOTE-158 study. Lancet Oncol..

[32] Marabelle A., Le D.T., Ascierto P.A., Di Giacomo A.M., De Jesus-Acosta A., Delord J.-P., Geva R., Gottfried M., Penel N., Hansen A.R. (2020). Efficacy of Pembrolizumab in Patients with Noncolorectal High Microsatellite Instability/Mismatch Repair–Deficient Cancer: Results from the Phase II KEYNOTE-158 Study. J. Clin. Oncol..

[33] Lemery S., Keegan P., Pazdur R. (2017). First FDA Approval Agnostic of Cancer Site—When a Biomarker Defines the Indication. N. Engl. J. Med..

[34] Mack E.K.M., Marquardt A., Langer D., Ross P., Ultsch A., Kiehl M.G., Mack H.I.D., Haferlach T., Neubauer A., Brendel C. (2019). Comprehensive genetic diagnosis of acute myeloid leukemia by next-generation sequencing. Haematologica.

[35] Wheler J.J., Janku F., Naing A., Li Y., Stephen B., Zinner R., Subbiah V., Fu S., Karp D., Falchook G.S. (2016). Cancer Therapy Directed by Comprehensive Genomic Profiling: A Single Center Study. Cancer Res..

[36] Bertucci F., Gonçalves A., Guille A., Adelaïde J., Garnier S., Carbuccia N., Billon E., Finetti P., Sfumato P., Monneur A. (2021). Prospective high-throughput genome profiling of advanced cancers: Results of the PERMED-01 clinical trial. Genome Med..

[37] Réda M., Richard C., Bertaut A., Niogret J., Collot T., Fumet J.D., Blanc J., Truntzer C., Desmoulins I., Ladoire S. (2020). Implementation and use of whole exome sequencing for metastatic solid cancer. eBioMedicine.

[38] Karczewski K.J., Francioli L.C., Tiao G., Cummings B.B., Alföldi J., Wang Q., Collins R.L., Laricchia K.M., Ganna A., Birnbaum D.P. (2020). The mutational constraint spectrum quantified from variation in 141,456 humans. Nature.

[39] Chakravarty D., Gao J., Phillips S., Kundra R., Zhang H., Wang J., Rudolph J.E., Yaeger R., Soumerai T., Nissan M.H. (2017). OncoKB: A Precision Oncology Knowledge Base. JCO Precis. Oncol..

[40] Griffith M., Spies N.C., Krysiak K., McMichael J.F., Coffman A.C., Danos A.M., Ainscough B.J., Ramirez C.A., Rieke D.T., Kujan L. (2017). CIViC is a community knowledgebase for expert crowdsourcing the clinical interpretation of variants in cancer. Nat. Genet..

[41] Patterson S.E., Liu R., Statz C.M., Durkin D., Lakshminarayana A., Mockus S.M. (2016). The clinical trial landscape in oncology and connectivity of somatic mutational profiles to targeted therapies. Hum. Genom..

[42] Landrum M.J., Lee J.M., Riley G.R., Jang W., Rubinstein W.S., Church D.M., Maglott D.R. (2013). ClinVar: Public archive of relationships among sequence variation and human phenotype. Nucleic Acids Res..

[43] Landrum M.J., Chitipiralla S., Brown G.R., Chen C., Gu B., Hart J., Hoffman D., Jang W., Kaur K., Liu C. (2020). ClinVar: Improvements to accessing data. Nucleic Acids Res..

[44] ClinGen/CGC/VICC SOP for the Classification of Pathogenicity of Somatic Variants in Cancer (Oncogenicity). https://cancervariants.org/research/standards/onc_path_sop/.

[45] Mateo J., Chakravarty D., Dienstmann R., Jezdic S., Gonzalez-Perez A., Lopez-Bigas N., Ng C., Bedard P., Tortora G., Douillard J.-Y. (2018). A framework to rank genomic alterations as targets for cancer precision medicine: The ESMO Scale for Clinical Actionability of molecular Targets (ESCAT). Ann. Oncol..

[46] Leichsenring J., Horak P., Kreutzfeldt S., Heining C., Christopoulos P., Volckmar A., Neumann O., Kirchner M., Ploeger C., Budczies J. (2019). Variant classification in precision oncology. Int. J. Cancer.

[47] Schildhaus H.U. (2018). Predictive Value of PD-L1 Diagnostics.

[48] Mock A., Heilig C.E., Kreutzfeldt S., Huebschmann D., Heining C., Schröck E., Brors B., Stenzinger A., Jäger D., Schlenk R. (2019). Community-driven development of a modified progression-free survival ratio for precision oncology. ESMO Open.

[49] Colomer R., Mondejar R., Romero-Laorden N., Alfranca A., Sanchez-Madrid F., Quintela-Fandino M. (2020). When should we order a next generation sequencing test in a patient with cancer?. EClinicalMedicine.

[50] Ciliberto G., Allegretti M., Babini G., Baldassarre G., Botti G., Bucci G., Buglioni S., Calistri D., Criscitiello C., Curigliano G. (2020). Linee Guida per l’istituzione e la Gestione dei Molecular Tumor Board Negli Istituti di Alleanza Contro il Cancro.

[51] Hoefflin R., Geißler A.L., Fritsch R., Claus R., Wehrle J., Metzger P., Reiser M., Mehmed L., Fauth L., Heiland D.H. (2018). Personalized Clinical Decision Making Through Implementation of a Molecular Tumor Board: A German Single-Center Experience. JCO Precis. Oncol..

[52] Heydt C., Wölwer C.B., Camacho O.V., Wagener-Ryczek S., Pappesch R., Siemanowski J., Rehker J., Haller F., Agaimy A., Worm K. (2021). Detection of gene fusions using targeted next-generation sequencing: A comparative evaluation. BMC Med. Genom..

[53] Robinson D.R., Wu Y.-M., Lonigro R.J., Vats P., Cobain E., Everett J., Cao X., Rabban E., Kumar-Sinha C., Raymond V. (2017). Integrative clinical genomics of metastatic cancer. Nature.

[54] Van Dijk E., van den Bosch T., Lenos K.J., El Makrini K., Nijman L.E., van Essen H.F.B., Lansu N., Boekhout M., Hageman J.H., Fitzgerald R.C. (2021). Chromosomal copy number heterogeneity predicts survival rates across cancers. Nat. Commun..

[55] Papaemmanuil E., Gerstung M., Bullinger L., Gaidzik V.I., Paschka P., Roberts N.D., Potter N.E., Heuser M., Thol F., Bolli N. (2016). Genomic Classification and Prognosis in Acute Myeloid Leukemia. N. Engl. J. Med..

[56] Döhner H., Wei A.H., Appelbaum F.R., Craddock C., DiNardo C.D., Dombret H., Ebert B.L., Fenaux P., Godley L.A., Hasserjian R.P. (2022). Diagnosis and Management of AML in Adults: 2022 ELN Recommendations from an International Expert Panel. http://ashpublications.org/blood/article-pdf/doi/10.1182/blood.2022016867/1906555/blood.2022016867.pdf.

[57] Dalens L., Niogret J., Kaderbhai C.G., Boidot R. (2022). Is There a Role for Large Exome Sequencing in the Management of Metastatic Non-Small Cell Lung Cancer: A Brief Report of Real Life. Front. Oncol..

[58] Heinrich K., Miller-Phillips L., Ziemann F., Hasselmann K., Rühlmann K., Flach M., Biro D., von Bergwelt-Baildon M., Holch J., Herold T. (2022). Lessons learned: The first consecutive 1000 patients of the CCCMunichLMU Molecular Tumor Board. J. Cancer Res. Clin. Oncol..

[59] Chakravarty D., Johnson A., Sklar J., Lindeman N.I., Moore K., Ganesan S., Lovly C.M., Perlmutter J., Gray S.W., Hwang J. (2022). Somatic Genomic Testing in Patients with Metastatic or Advanced Cancer: ASCO Provisional Clinical Opinion. J. Clin. Oncol..

[60] Mosele F., Remon J., Mateo J., Westphalen C., Barlesi F., Lolkema M., Normanno N., Scarpa A., Robson M., Meric-Bernstam F. (2020). Recommendations for the use of next-generation sequencing (NGS) for patients with metastatic cancers: A report from the ESMO Precision Medicine Working Group. Ann. Oncol..

[61] Inagaki C., Maeda D., Hatake K., Sato Y., Hashimoto K., Sakai D., Yachida S., Nonomura N., Satoh T. (2021). Clinical Utility of Next-Generation Sequencing-Based Panel Testing under the Universal Health-Care System in Japan: A Retrospective Analysis at a Single University Hospital. Cancers.

[62] Langmead B., Salzberg S.L. (2012). Fast gapped-read alignment with Bowtie 2. Nat. Methods.

[63] Li H. (2011). A statistical framework for SNP calling, mutation discovery, association mapping and population genetical parameter estimation from sequencing data. Bioinformatics.

[64] Liao Y., Smyth G.K., Shi W. (2014). feature Counts: An efficient general purpose program for assigning sequence reads to genomic features. Bioinformatics.

[65] Quinlan A.R. (2014). BEDTools: The Swiss-Army Tool for Genome Feature Analysis. Curr. Protoc. Bioinform..

[66] Benjaminit Y., Hochberg Y. (1995). Controlling the False Discovery Rate: A Practical and Powerful Approach to Multiple Testing. J. R. Stat. Soc. B.

[67] Cheung V.G., Nowak N., Jang W., Kirsch I.R., Zhao S., Chen X.N., Furey T.S., Kim U.J., Kuo W.L., Olivier M. (2001). Integration of cytogenetic landmarks into the draft sequence of the human genome. Nature.

[68] (2020). An International System for Human Cytogenomic Nomenclature. https://www.karger.com/Book/Home/279152.

[69] Bailey M.H., Tokheim C., Porta-Pardo E., Sengupta S., Bertrand D., Weerasinghe A., Colaprico A., Wendl M.C., Kim J., Reardon B. (2018). Comprehensive Characterization of Cancer Driver Genes and Mutations. Cell.

[70] Anai S., Takeshita M., Ando N., Ikematsu Y., Mishima S., Ishida K., Inoue K. (2016). A Case of Lung Adenocarcinoma Resistant to Crizotinib Harboring a Novel EML4-ALK Variant, Exon 6 of EML4 Fused to Exon 18 of ALK. J. Thorac. Oncol..

[71] Mateo J., Carreira S., Sandhu S., Miranda S., Mossop H., Perez-Lopez R., Nava Rodrigues D., Robinson D., Omlin A., Tunariu N. (2015). DNA-Repair Defects and Olaparib in Metastatic Prostate Cancer. N. Engl. J. Med..

[72] Kopetz S., Grothey A., Yaeger R., Van Cutsem E., Desai J., Yoshino T., Wasan H., Ciardiello F., Loupakis F., Hong Y.S. (2019). Encorafenib, Binimetinib, and Cetuximab in BRAF V600E–Mutated Colorectal Cancer. N. Engl. J. Med..

[73] Okamura R., Kato S., Lee S., Jimenez R.E., Sicklick J.K., Kurzrock R. (2020). ARID1A alterations function as a biomarker for longer progression-free survival after anti-PD-1/PD-L1 immunotherapy. J. Immunother. Cancer.

[74] Bentzien F., Zuzow M., Heald N., Gibson A., Shi Y., Goon L., Yu P., Engst S., Zhang W., Huang D. (2013). In vitro and in vivo activity of cabozantinib (XL184), an inhibitor of RET, MET, and VEGFR2, in a model of medullary thyroid cancer. Thyroid.

[75] Dummer R., Ascierto P.A., Gogas H.J., Arance A., Mandala M., Liszkay G., Garbe C., Schadendorf D., Krajsova I., Gutzmer R. (2018). Encorafenib plus binimetinib versus vemurafenib or encorafenib in patients with BRAF -mutant melanoma (COLUMBUS): A multicentre, open-label, randomised phase 3 trial. Lancet Oncol..

[76] Sabatier R., Lopez M., Guille A., Billon E., Carbuccia N., Garnier S., Adelaide J., Extra J.-M., Cappiello M.-A., Charafe-Jauffret E. (2019). High Response to Cetuximab in a Patient With EGFR-Amplified Heavily Pretreated Metastatic Triple-Negative Breast Cancer. JCO Precis. Oncol..

[77] Zhu H., Wang C., Wang J., Chen D., Deng J., Deng J., Fan J., Badakhshi H., Huang X., Zhang L. (2018). A subset of esophageal squamous cell carcinoma patient-derived xenografts respond to cetuximab, which is predicted by high EGFR expression and amplification. J. Thorac. Dis..

[78] Kiyuna T., Murakami T., Tome Y., Igarashi K., Kawaguchi K., Miyake K., Miyake M., Li Y., Nelson S.D., Dry S.M. (2018). Doxorubicin-resistant pleomorphic liposarcoma with PDGFRA gene amplification is targeted and regressed by pazopanib in a patient-derived orthotopic xenograft mouse model. Tissue Cell.

[79] Camidge D.R., Dziadziuszko R., Peters S., Mok T., Noe J., Nowicka M., Gadgeel S.M., Cheema P., Pavlakis N., de Marinis F. (2019). Updated Efficacy and Safety Data and Impact of the EML4-ALK Fusion Variant on the Efficacy of Alectinib in Untreated ALK-Positive Advanced Non–Small Cell Lung Cancer in the Global Phase III ALEX Study. J. Thorac. Oncol..

