# Supplementary material for: Combined Focused Next-Generation Sequencing Assays to Guide Precision Oncology in Solid Tumors: A Retrospective Analysis from an Institutional Molecular Tumor Board
Source: Cancers (Basel). 2022 Sep 12;14(18):4430. doi: 10.3390/cancers14184430 (PMC9496918; doi:10.3390/cancers14184430)
Supplement: Supplementary file 1 [file cancers-14-04430-s001.zip › Tarawneh et al_Supplementary materials.pdf]

<u>Supplementary Materials</u>

Combined focused Next-Generation Sequencing assays to guide precision oncology in solid tumors: a retrospective analysis from an institutional Molecular Tumor Board

Thomas S. Tarawneh et al.

Contents

Supplementary Methods 1
Supplementary Table S1.......... 3
Supplementary Table S2.......... 5
Supplementary Table S3.......... 8
Supplementary Figure S1 16
Supplementary Figure S2 18
Supplementary Table S4.......... 19
Supplementary References 20

Supplementary Methods

Calculation of numerical karyotypes by CORIANDR
CORIANDR is a read-depth based tool for numerical karyotype and CNA estimation from the raw reads of low coverage WGS sequencing. We use a panel of normals (PON) to identify and reduce technical artifacts. The samples for the panel of normals were obtained and sequenced under the same conditions as the tumor samples. R Studio v3.6.3 was used for statistical analysis and reports in markdown. For visualization knitr v1.30, markdown v1.1, rmarkdown v2.6, tinytex v0.28 packages in R Studio were used.

Data preparation
The sequencing data from Illumina MiSeq was aligned to the reference genome with Bowtie 2 v2.3.5.1 [1] in paired-end mode. We used version GRCh38.p13 of the human genome from Genome Reference Consortium as reference genome for alignment. The data was converted to BAM format using samtools v1.10 [2]. We count the reads in the non-overlapping megabase sized bins using featureCounts v2.0.0 [3].

Generation of a panel of normals
To generate a panel of normals, the reads of all PON samples are summed up after counting per bin. The reads on autosomal chromosomes are used for the analysis of both genders, while the reads on the X and Y chromosomes are only used as a statistical reference for the same gender. In the next step, the PON is normalized by median per library size and per megabase for the whole genome, including X and Y chromosomes.
To improve the quality of the PON and to minimize technical artefacts, we identify the bins with a GC content that deviates from the expected value for the human genome. We determined the GC content of the reference genome using Bedtools v2.27.1 [4]. Bins with a GC-content less than the 0.275 percentile genome-wide assumed normal distribution were considered to be extreme and masked from the PON. Additionally, bins with the highest 1% variance genome-wide were masked from the PON.

A report for creating a panel of normals is then generated with the plots for sequencing depth of individual PON subjects, normalizing PON with a scaling factor equal to 2 (ploidy) for autosomes and equal to 1 for X and Y in male individuals and PON chromosomal overview with masking bins.

Estimation of calculated karyotype and CNA

In the first step, the data of the tumor sample are normalized by the median sequencing depth per bin. Thereafter, we used standardization with calculation of the pseudo z-values of the distribution of the bins. Later they will be compared with the theoretical normal distribution.

$$\frac{x_i - 1}{\sigma_{(X)}} = Z_i,$$

where x_i represents the reads in the bin, $\sigma_{(X)}$ is the standard deviation of the bin estimated from the PON, Z_i is the pseudo z-score of the bin.

In addition, we tested pseudo z-scores against a normal distribution with parameters of the PON in a two-tailed test. The obtained p-values were adjusted using the Benjamini-Hochberg method [5] in control the false discovery rate.

$$Z_i \sim N(\overline{z_i^{PON}}, \sigma_i{}^{PON})$$

where Z_i is the z-score of the bin in the sample derived from a normal distribution, *N*, with mean z-score and variance of this bin in the PON.

In consideration of the adjusted p-values, the deviating bins are calculated. A deletion in a bin is detected if the normalized value for that bin is below the median for all normalized PON samples with a significance level of $\alpha = 0.05$. An amplification leads to a value above the median.

For the estimation of the calculated karyotype, we used the genomic coordinates of the G-bands from the cytogenetic landmarks [6]. Since the G-bands are longer than one megabase we make sure that the start and end points of the bins overlap with the start and end positions of the bands as much as possible. We determined the start point of a bin that was as close as possible to the start position of a band. A bin with the closest possible to end point had to correspond the end point of a band. The masked bins were not included here. If half of all bins in a band have the status "deleted", that band is also considered deleted. The decision value of half of the bins also applies to the band amplification. All other bins are classified as non-aberrant.

To facilitate the interpretation of the report for CORIANDR users, we decided to group the bands together according to the ISCN nomenclature [7]. To merge the bands, we make sure whether they are on the same chromosome and contiguous and whether they have the same aberration status. Examining the coordinates for the G-bands we then define the loss or amplification of whole chromosome arms or whole chromosomes. If more than 80% of the G-bands of a chromosome arm or a whole chromosome show aberrations of the same type, we call it deleted or amplified.

Another feature of CORIANDR is the copy number alterations estimation of the individual genes. By examination the localization of the relevant genes in the aberrant bins, genetic mutations can be detected. The investigated genes are based on the list of the WHO classification of Acute Myeloid Leukemia [8] and the cancer driver genes [9]. If the positions of genes found in the aberrant bins, are also considered as aberrant in the report.

Supplementary Table S1.
Target regions of the VariantPlex Solid Tumor Panel (ArcherDx/Invitae)

Gene	Accession	Exon number	SSVs	CNA
ABL1	NM_005157	4,5,6,7	yes	yes
AKT1	NM_005163	3,6	yes	no
ALK	NM_004304	21,22,23,25	yes	yes
APC	NM_000038	16	yes	yes
ATM	NM_000051	8,9,12,17,26,34,35,36,39,50,54,55,56,59,61,63	yes	yes
AURKA	NM_003600	2,5,6,7,8	yes	yes
BRAF	NM_004333	11,15	yes	no
CCND1	NM_053056	na	no	yes
CCNE1	NM_001238	3,4,5,6,7,8,10,12	yes	yes
CDH1	NM_004360	1,2,3,4,5,6,7,8,9,10,11,12,13,14,15,16	yes	yes
CDK4	NM_000075	2,3,4,5,6,7	yes	yes
CDKN2A	NM_000077	1,2,3	yes	yes
CSF1R	NM_005211	7,22	yes	no
CTNNB1	NM_001904	3	yes	no
DDR2	NM_006182	12,13,14,15,16,17,18	yes	yes
EGFR	NM_005228	3,7,15,18,19,20,21	yes	yes
ERBB2	NM_004448	10,19,20,21,24	yes	yes
ERBB3	NM_001982	2,3,7,8	yes	yes
ERBB4	NM_005235	3,4,6,7,8,9,15,23	yes	yes
ESR1	NM_000125	8	yes	no
EZH2	NM_004456	16	yes	no
FBXW7	NM_018315	1,2,3,4,5,6,7,8,9,10,11	yes	yes
FGFR1	NM_015850	4,7,8,13,15,17	yes	yes
FGFR2	NM_000141	7,9,12,14	yes	yes
FGFR3	NM_000142	7,8,9,14,15,16,18	yes	yes
FLT3	NM_004119	11,14,16,20	yes	yes
FOXL2	NM_023067	1	yes	no
GNA11	NM_002067	5	yes	no
GNAQ	NM_002072	4,5	yes	no
GNAS	NM_000516	6,7,8,9	yes	yes
H3F3A	NM_002107	2	yes	no
HNF1A	NM_000545	3,4	yes	no
HRAS	NM_005343	2,3	yes	no
IDH1	NM_005896	3,4	yes	no
IDH2	NM_002168	4	yes	no
JAK2	NM_004972	11,13,14,16,19	yes	yes
JAK3	NM_000215	4,13,16	yes	yes
KDR	NM_002253	6,7,11,19,21,26,27,30	yes	yes

KIT	NM_000222	2,8,9,10,11,13,14,15,17,18	yes	yes
KRAS	NM_004985	2,3,4,5	yes	yes
MAP2K1	NM_002755	2,3	yes	no
MDM2	NM_002392	2,3,4,6,8*,10	no	yes
MET	NM_000245	2,11,14,16,19,21	yes	yes
MYC	NM_002467	na	no	yes
MYCN	NM_005378	na	no	yes
MLH1	NM_000249	12	yes	no
MPL	NM_005373	10	yes	no
NOTCH1	NM_017617	25,26,27,34	yes	yes
NPM1	NM_002520	11	yes	no
NRAS	NM_002524	2,3,4,5	yes	yes
PDGFRA	NM_006206	12,14,15,18,23	yes	yes
PIK3CA	NM_006218	2,5,7,8,10,14,19,21	yes	yes
PIK3R1	NM_181504	1,2,3,4,5,6,7,8,9,10	yes	yes
PTEN	NM_000314	1,2,3,4,5,6,7,8,9	yes	yes
PTPN11	NM_002834	3,13	yes	no
RB1	NM_000321	4,6,10,11,14,17,18,20,21,22*	yes	yes
RET	NM_020630	10,11,13,14,15,16	yes	yes
RHOA	NM_001664	2,3	yes	no
ROS1	NM_002944	38	yes	no
SMAD4	NM_005359	2,3,4,5,6,7,8,9,10,11,12	yes	yes
SMARCB1	NM_003073	2,4,5,9	yes	yes
SMO	NM_005631	3,5,6,9,11	yes	yes
SRC	NM_005417	14	yes	no
STK11	NM_000455	1,2,3,4,5,6,7,8,9	yes	yes
TERT	NM_198253	Promoter, 1	yes	no
TP53	NM_000546	1,2,3,4,5,6,7,8,9,10,11	yes	yes
VHL	NM_000551	1,2,3	yes	yes

*Red asterisk denotes partial coverage of that exon for SSVs

Supplementary Table S2.
Target genes of the FusionPlex Solid Tumor Panel (ArcherDx/Invitae)

Gene	Accession	Exon	Assay type	Direction
AKT3	NM_005465	1, 2, 3	Fusion	5’
ALK	NM_004304	19, (intron 19), 20, 21, 22	Fusion	5’
ARHGAP26	NM_015071	2, 10, 11, 12	Fusion	5’
AXL	NM_021913	19, 20	Fusion	3’
BRAF	NM_004333	7, 8, 9, 10, 11, 12	Fusion	5’
BRAF	NM_004333	7, 8	Fusion	3’
BRAF	NM_004333	V600Ei	Mutation	N/A
BRD3	NM_007371	9, 10, 11, 12	Fusion	3’
BRD4	NM_014299	10, 11	Fusion	3’
EGFR	NM_005228	7, 9, 16, 20	Fusion	5’
EGFR	NM_005228	8 (2-7 exon skipping event)	Fusion	N/A
EGFR	NM_005228	24, 25	Fusion	3’
ERG	NM_004449	2, 3, 4, 5, 6, 7, 8, 9, 10, 11	Fusion	5’
ESR1	NM_001122742	3, 4, 5, 6	Fusion	3’
ETV1	NM_004956	3, 4, 5, 6, 7, 8, 9, 10, 11,12, 13	Fusion	5’
ETV4	NM_001986	2, 4, 5, 6, 7, 8, 9, 10	Fusion	5’
ETV5	NM_004454	2, 3, 7, 8, 9	Fusion	5’
ETV6	NM_001987	2, 3, 5, 6, 7	Fusion	5’
ETV6	NM_001987	1, 2, 3, 4, 5, 6	Fusion	3’
EWSR1	NM_005243	4, 5, 6, 7, 8, 9, 10, 11, 12, 13, 14	Fusion	3’
FGFR1	NM_015850	2, 8, 9, 10	Fusion	5’
FGFR1	NM_015850	17	Fusion	3’
FGFR2	NM_000141	2, 8, 9, 10	Fusion	5’
FGFR2	NM_000141	17	Fusion	3’
FGFR3	NM_000142	8, 9, 10	Fusion	5’
FGFR3	NM_000142	17, Intron 17	Fusion	3’
FGR	NM_005248	2	Fusion	5’
INSR	NM_000208	12, 13, 14, 15, 16, 17, 18, 19	Fusion	5’

INSR	NM_000208	20, 21, 22	Fusion	3’
MAML2	NM_032427	2, 3	Fusion	5’
MAST1	NM_014975	7, 8, 9, 18, 19, 20, 21	Fusion	5’
MAST2	NM_015112	2, 3, 5, 6	Fusion	5’
MET	NM_000245	13, 15 (exon 14 skipping event)	Fusion	N/A
MET	NM_000245	2, 13, 14, 16	Fusion	5’
MSMB	NM_002443	2, 3, 4	Fusion	3’
MUSK	NM_005592	7, 8, 9, 11, 12, 13, 14	Fusion	5’
MYB	NM_001130173	7, 8, 9, 11, 12, 13, 14, 15, 16	Fusion	3’
NOTCH1	NM_017617	26, 27, 28, 29 (internal deletion exons 3-27)	Fusion	5’
NOTCH1	NM_017617	2, 4, 29, 30, 31	Fusion	3’
NOTCH2	NM_024408	26, 27, 28	Fusion	5’
NOTCH2	NM_024408	5, 6, 7	Fusion	3’
NRG1	NM_004495	1, 2, 3, 6	Fusion	5’
NTRK1	NM_002529	8, 10, 11, 12, 13	Fusion	5’
NTRK2	NM_006180	11, 12, 13, 14, 15, 16, 17	Fusion	5’
NTRK3	NM_002530	13, 14, 15, 16	Fusion	5’
NTRK3	NM_001007156	15	Fusion	5’
NUMBL	NM_004756	3	Fusion	5’
NUTM1	NM_175741	3	Fusion	5’
PDGFRA	NM_006206	7 (exon 8 deletion)	Mutation	N/A
PDGFRA	NM_006206	10, 11, 12, 13, 14	Fusion	5’
PDGFRA	NM_006206	T674I, D842V	Mutation	N/A
PDGFRB	NM_002609	8, 9, 10, 11, 12, 13, 14	Fusion	5’
PIK3CA	NM_006218	2	Fusion	5’
PKN1	NM_002741	10, 11, 12, 13	Fusion	5’
PPARG	NM_015869	1, 2, 3	Fusion	5’
PRKCA	NM_002737	4, 5, 6	Fusion	5’
PRKCB	NM_002738	3	Fusion	5’
RAF1	NM_002880	4, 5, 6, 7, 9, 10, 11, 12	Fusion	5’

RAF1	NM_002880	4, 5, 6, 7, 9	Fusion	3’
RELA	NM_021975	3, 4	Fusion	5’
RET	NM_020630	8, 9, 10, 11, 12, 13	Fusion	5’
ROS1	NM_002944	31, 32, 33, 34, 35, 36, 37	Fusion	5’
RSPO2	NM_178565	1, 2	Fusion	5’
RSPO3	NM_032784	2	Fusion	5’
TERT	NM_198253	2	Fusion	5’
TFE3	NM_006521	2, 3, 4, 5, 6, 7, 8	Fusion	5’
TFE3	NM_006521	2, 3, 4, 5, 6	Fusion	3’
TFEB	NM_007162	1, 2	Fusion	5’
THADA	NM_022065	28	Fusion	3’
TMPRSS2	NM_001135099	1	Fusion	3’
TMPRSS2	NM_005656	1, 2, 3, 4, 5, 6	Fusion	3’

Supplementary Table S3.

Target genes of the FusionPlex Pan Solid Tumor Panel (ArcherDx/Invitae)

Gene	Accession	Exon	Assay type	Direction
ACVR2A	NM_001616	1, 2, 3	Fusion	5'
AKT1	NM_005163	2, 3, 4, 5, mid-exon5	Fusion	5'
AKT2	NM_001626	2*, 5	Fusion	5'
AKT2	NM_001626	11	Fusion	3'
AKT3	NM_005465	2, 3, 4, 9	Fusion	5'
AKT3	NM_005465	6, 7, 8	Fusion	3'
ALK	NM_004304	2, 4, 6, 8, 10, 12,14, 16, 17, 18, 19, intron19, 20, mid-exon20, 21, 22, 23, 26	Fusion, ALK ATI, Internal deletion (ALKΔ2-17, ALKΔ2-3)	5'
ALK	NM_004304	1, 2	Internal deletion (ALKΔ2- 17, ALKΔ2-3)	3'
ALK	NM_004304	22, 23, 25	Mutation	p.P1153- p.C1156, p.F1174, p.L1196- p.S1206, p.G1269
AR	NM_001011645	1	Fusion	3'
AR	NM_000044	1, 2, 3, 4, 5, 6, 7, 8*	Fusion, ARv7	3'
ARHGAP26	NM_015071	2, 10, 11, 12	Fusion	5'
ARHGAP6	NM_006125	2	Fusion	5'
AXL	NM_021913	11	Fusion	5'
AXL	NM_021913	18, 19, mid-exon20, 20	Fusion	3'
BCOR	NM_017745	8	Fusion	5'
BCOR	NM_001123385	mid-exon2, 3, 4, mid-exon4, 5, 6, 7, 8, 9, 11, 15	Fusion, Internal Tandem Duplication	5'
BCOR	NM_001123385	2, 4, mid-exon4, 6, 7, mid-exon7, 10, 12, 14, 15	Fusion, Internal Tandem Duplication	3'
BRAF	NM_004333	2, 3, 4, 5, 7, 8, 9, 10, 11, 12, 15, 16	Fusion, Kinase Domain Duplication, BRAFΔ2-10, BRAFΔ4-10, BRAFΔ2-8, BRAFΔ3-8, BRAFΔ4-8	5'
BRAF	NM_004333	1, 2, 3, 7, 8, 10, 13, 14, 18	Fusion, Kinase Domain Duplication, BRAFΔ2-10, BRAFΔ4-10, BRAFΔ2-8, BRAFΔ3-8, BRAFΔ4-8	3'
BRAF	NM_004333	15	Mutation	p.V600
BRD3	NM_007371	9, 10, 11, 12	Fusion	3'
BRD4	NM_058243	2*	Fusion	5'

BRD4	NM_058243	10, 11, 12, 13, 14	Fusion	3'
CAMTA1	NM_015215	8, 9, mid-exon9, 10	Fusion	5'
CAMTA1	NM_015215	3	Fusion	3'
CCNB3	NM_033031	2*, 3, 4, 5, 6, mid-exon 6, 7	Fusion	5'
CCND1	NM_053056	1*, 2, 3, 4, 5	Fusion	5'
CCND1	NM_053056	1, 2, 3, 4, mid-exon5*	Fusion	3'
CCND1	NM_053056	5*	Fusion	3'
CCND1	NM_053056	1	Mutation	p.E36-p.C47
CD274	NM_014143	7	Expression	5'
CD274	NM_014143	2, 3, 4, 5	Expression	3'
CIC	NM_015125	12	Fusion	5'
CIC	NM_015125	14, 15, 16, 17, 18, mid-exon19, 19, mid-exon20,20*	Fusion	3'
CRTC1	NM_015321	1, 2, 3, 4	Fusion	3'
CSF1	NM_000757	2, 3, 4, 5, 6	Fusion	5'
CSF1	NM_000757	5, 6, 7, 8*, mid-exon9*	Fusion	3'
CSF1	NM_172212	9*	Fusion	3'
CSF1R	NM_005211	11, 12, 13	Fusion	5'
CTNNB1	NM_001904	3	Mutation	p.D32-p.S37
DNAJB1	NM_006145	1, 2	Fusion	3'
EGF	NM_001963	16, 17, 18, 19	Fusion	5'
EGFR	NM_005228	7, 8, 9, 14, 15, 16, 17, 18, 19, 20	Fusion, Exon 2-7 Skipping (EGFRvIII),Kinase Domain Duplication	5'
EGFR	NM_005228	1, 24, 25, mid-exon25, 26	Fusion, Exon 2-7 Skipping (EGFRvIII),Kinase Domain Duplication	3'
EGFR	NM_005228	18, 19, 20, 21	Mutation	p.E709-p.G719, p.E746-p.L760, p.V774- p.G796, p.L858-p.L861
EPC1	NM_025209	9, 10, 11	Fusion	3'
ERBB2	NM_004448	4, 5, 13, 15, 17	Fusion, Exon 16 skipping (Δ16HER)	5'
ERBB2	NM_004448	15, 23, 24, 25, mid-exon26, 26	Fusion, Exon 16 skipping (Δ16HER)	3'
ERBB2	NM_004448	8, 20	Mutation	p.G309-p.S310, p.Y772-p.P780, p.C805
ERBB4	NM_005235	2, 3, 4, 14, 15, 16, 17, 18, 23	Fusion	5'

ERG	NM_004449	2*, 3*, 4, 5, 6, 7, 8, 9, 10, 11	Fusion	5'
ESR1	NM_000125	5, 6, 7, 8	Fusion	5'
ESR1	NM_000125	1, 2, 3, 4, 5, 6, 7	Fusion	3'
ESRRA	NM_004451	2, 3	Fusion	3'
ETV1	NM_004956	3, 4, 5, 6, 7, 8, 9, 10, 11, 12, 13	Fusion	5'
ETV4	NM_001986	2, 3, 4, 5, 6, 7, 8, 9, 10	Fusion	5'
ETV5	NM_004454	2*, 3, 7, 8, 9	Fusion	5'
ETV6	NM_001987	2, 3, 4, 5, 6, 7	Fusion	5'
ETV6	NM_001987	1, 2, 3, 4, 5, 6	Fusion	3'
EWSR1	NM_005243	8	Fusion	5'
EWSR1	NM_005243	4, 5, 6, 7, 8, 9,10, 11, 12, 13, 14	Fusion	3'
FGF1	NM_00800	mid-exon 2, 2	Fusion	5'
FGFR1	NM_015850	2*, 3, 4, 5, 6, 7, 8, 9, 10, 11, 17	Fusion, Kinase Domain Duplication	5'
FGFR1	NM_015850	12, 17	Fusion, Kinase Domain Duplication	3'
FGFR1	NM_023110	4, 13, 14	Mutation	p.T141, p.V561, p.K656
FGFR2	NM_000141	2*, 3, 5, 6, 7, 8, 9, 10	Fusion	5'
FGFR2	NM_000141	16, 17, 18	Fusion	3'
FGFR2	NM_000141	7, 9, 12, 13, 14	Mutation	p.S252- p.P253, p.G305, p.Y375-V395, p.I547-p.N549, p.V564, p.A648-p.K659
FGFR3	NM_000142	3, 5, 8, 9, 10, 11, 12, 13, 14	Fusion	5'
FGFR3	NM_000142	16, 17, intron17,mid-exon18	Fusion	3'
FGFR3	NM_000142	7, 9, 13, 14, 16	Mutation	p.R248-p.S249, p.G370-p.R399, p.V555, p.D641-p.K650, p.G697-p.K715
FGR	NM_005248	2*, 3	Fusion	5'
FOS	NM_005252	mid-exon4	Fusion	3'
FOSB	NM_006732	1*, mid-exon1*,1, 2	Fusion	5'
FOXO1	NM_002015	1*, 2, 3*	Fusion	5'
FOXO1	NM_002015	1*, 2*, 3*	Fusion	3'
FOXO4	NM_005938	2, mid-exon2, 3	Fusion	5'
FOXR2	-	cryptic upstreamexon2,3 (chrX:55562068, chrX:55634636)	Fusion	5’
FUS	NM_004960	3, 4, 5, mid-exon6, 6, 7, 8, 9, 10, 11, 13, 14	Fusion	3'

GLI1	NM_005269	4, 5, 6, 7	Fusion	5'
GLI1	NM_005269	4, 5, mid-exon5, 6, 7	Fusion	3'
GRB7	NM_005310	10, 11, 12	Fusion	5'
HMGA2	NM_003483	1, 2, 3, 4, mid-exon5*, 5*	Fusion	3'
HRAS	NM_005343	2, 3, 4	Mutation	p.G12- p.G13, p.Q61, p.K117, p.A146
IDH1	NM_005896	4	Mutation	p.R132
IDH2	NM_002168	4	Mutation	p.R140, p.R172
IGF1R	NM_000875	13, 14, 15	Fusion	5'
INSR	NM_000208	2, 12, 13, 14, 15, 16, 17, 18, 19	Fusion	5'
INSR	NM_000208	20, 21, 22	Fusion	3'
JAK2	NM_004972	6, 7, 8, 9, 10, 11, 12, 13, 14, 15, 16, 17, 18, 19, 20	Fusion	5'
JAK2	NM_004972	9, 10, 11, 12, 22	Fusion	3'
JAK2	NM_004972	12, 14, 16, 19, 20	Mutation	p.F537-p.F547, p.V617-p.C618, p.L681-p.R683, p.L855, p.V863
JAK3	NM_000215	10, 11, 12, 17, 18, 19	Fusion	5'
JAK3	NM_000215	23	Fusion	3'
JAK3	NM_000215	11, 17	Mutation	p.M511, p.S789
GLI1	NM_005269	4, 5, 6, 7	Fusion	5'
GLI1	NM_005269	4, 5, mid-exon5, 6, 7	Fusion	3'
GRB7	NM_005310	10, 11, 12	Fusion	5'
JAZF1	NM_175061	2, 3, 4	Fusion	3'
KIT	NM_000222	8	Fusion	5'
KIT	NM_000222	1	Fusion	3'
KRAS	NM_004985	2, 3, 4	Mutation	p.G12- p.G13, p.Q61, p.K117, p.A146
MAML2	NM_032427	2, 3	Fusion	5'
MAML2	NM_032427	2	Fusion	3'
MAP2K1	NM_002755	2	Fusion	5'
MAP2K1	NM_002755	2	Mutation	p.F53-p.D67
MAST1	NM_014975	7, 8, 9, 18, 19, 20, 21	Fusion	5'
MAST2	NM_015112	2, 3, 5, 6	Fusion	5'
MAST2	NM_015112	15, 16, 17	Fusion	3'
MBTD1	NM_017643	3*	Fusion	5'
MBTD1	NM_017643	15, 16, 17	Fusion	3'
MDM2	NM_002392	5, 9	Fusion, Expression	5'
MDM2	NM_002392	2, 4, 6, 8, 10	Fusion,	3'

			Expression	
MEAF6	NM_001270875	4, 5	Fusion	3'
MET	NM_000245	2, 4, 5, 6, 13, 14, 15, 16, 17, 21	Fusion, Exon 14 Skipping (METΔex14)	5'
MET	NM_000245	2, 13	Fusion, Exon 14 Skipping (METΔex14)	3'
MGEA5	NM_012215	4, 5, 6, 7, 8, 9, 12, 13, 14, 15	Fusion, Expression	5'
MKL2	NM_014048	11, 12, 13	Fusion	5'
MN1	NM_002430	1, 2	Fusion	3'
MSMB	NM_002443	2, 3, 4	Fusion	3'
MUSK	NM_005592	7, 9, 10, 12, 13, 14, 15	Fusion	5'
MYB	NM_001130173	7, 8, 9, 11, 12, 13, 14, 15, 16	Fusion	3'
MYBL1	NM_001080416	8, 9, mid-exon10, 10, 11, 12, 13, 14, 15	Fusion	3'
MYC	NM_002467	1, 2	Expression	3'
MYC	NM_002467	1*, mid-exon1*,2, 3	Fusion, Expression	5'
MYOD1	NM_002478	1	Mutation	p.L122, full CDS coverage for mutation detection
NCOA1	NM_147223	11, 12, 13, 14,15	Fusion	5'
NCOA2	NM_006540	11, 12, 13, 14, intron14, 15, 16, 22	Fusion	5'
NCOA2	NM_006540	14	Fusion	3'
NCOA3	NM_006534	2*, 13, 14, 15, 16	Fusion	5'
NCOA3	NM_006534	20	Fusion	3'
NFATC2	NM_012340	2, 3, 9, 10	Fusion	5'
NFE2L2	NM_006164	1, 2, 3, 4, 5	Exon skipping, Fusion	5'
NFIB	NM_001369458	10, 11	Fusion	5'
NFIB	NM_005596	9*, mid-exon 9	Fusion	5'
NFIB	NM_005596	2	Fusion	3'
NOTCH1	NM_017617	2, 4, 24, 29, 30, 31	Fusion	3'
NOTCH1	NM_017617	5, 24, 25, 26, 27, 28, 29	Fusion,Exon Skipping(NOTCH1Δ2-27,NOTCH1Δ21-27, NOTCH1Δ3-27, NOTCH1Δ3-28)	5'
NOTCH2	NM_024408	24, 25, 26, 27, 28, 29	Fusion	5'
NOTCH2	NM_024408	5, 6, 7	Fusion	3'
NR4A3	NM_173200	2*, 3*, 4, 5, 7, 9	Fusion, Expression	5'
NR4A3	NM_173200	8	Fusion, Expression	3'

NRAS	NM_002524	2, 3, 4	Mutation	p.G12-p.G13,p.Q61, p.K117, p.A146
NRG1	NM_001159996	1*, 3, 4, 5	Fusion	5'
NRG1	NM_004495	1, 2, 3, 4, 5, 6	Fusion	5'
NRG1	NM_013958	1*	Fusion	5'
NRG1	NM_013959	1*, 3	Fusion	5'
NRG1	NM_013962	1*	Fusion	5'
NRG1	NM_013962	1	Fusion	3'
NTRK1	NM_001007792	1, 2	Fusion	5'
NTRK1	NM_002529	1, 2, 3, 4, 5, 6, 7, 8, 9, 10, 11, 12, 13, 14	Fusion	5'
NTRK1	NM_002529	13, 14, 15, 16, 17	Mutation	Full Kinase Domain coverage for resistance mutations including p.G595
NTRK2	NM_006180	4, 5, 6, 7, 8, 9, 10, 11, 12, 13, 14, 15, 16, 17, 18	Fusion	5'
NTRK2	NM_006180	11, 14	Fusion	3'
NTRK2	NM_006180	16, 17, 18, 19, 20, 21	Mutation	Full Kinase Domain coverage for resistance mutations
NTRK3	NM_001007156	15	Fusion	5'
NTRK3	NM_002530	3, 4, 5, 6, 7, 8, 9, 10, 11, 12, 13, 14, 15, 16	Fusion	5'
NTRK3	NM_002530	13, 14, 15, 17	Fusion	3'
NTRK3	NM_002530	15, 16, 17, 18, 19	Mutation	Full Kinase Domain coverage for resistance mutation detection including p.F617, p.G623, p.G696
NUMBL	NM_004756	2, 3	Fusion	5'
NUTM1	NM_175741	2*, 3, mid-exon3, 4, 5, mid-exon6, 6	Fusion	5'
PAX3	NM_181459	2, 4, 8	Fusion, Expression	5'
PAX3	NM_181459	3, 5, 6, 7, 8	Fusion, Expression	3'
PAX8	NM_003466	3	Fusion	5'
PAX8	NM_003466	1*, 2, 6, 7, 8, 9, 10	Fusion	3'
PDGFB	NM_002608	2, 3	Fusion	5'
PDGFD	NM_025208	5, 6, 7	Fusion	5'
PDGFRA	NM_006206	10, 11, 12, mid-exon12, 13, 14, 15	Fusion,PDGFRAΔ 8,9	5'
PDGFRA	NM_006206	7	Fusion,PDGFRAΔ	3'

			8,9	
PDGFRA	NM_006206	15, 18	Mutation	p.T674, p.D842
PDGFRB	NM_002609	8, 9, 10, 11, 12, mid-exon12, 13, 14	Fusion	5'
PHF1	NM_024165	1*, 2	Fusion	5'
PHF1	NM_024165	10, 11, 12	Fusion	3'
PHKB	NM_000293	4	Fusion	3'
PIK3CA	NM_006218	2, 15	Fusion	5'
PIK3CA	NM_006218	2, 3, 5, 6, 8, 10, 14, 21	Mutation	p.E81K-p.G118D, p.L339-p.D350, p.G364R, p.E418-p.C420, p.E453-p.K468, p.P539-p.Q546, p.E726,p.Y1021-p.T1052
PKN1	NM_002741	10, 11, 12, 13	Fusion	5'
PLAG1	NM_002655	1, 2, 3, 4	Fusion	5'
PPARG	NM_015869	1, 2, 3	Fusion	5'
PRDM10	NM_020228	13, 14	Fusion	5'
PRKACA	NM_002730	2	Fusion	5'
PRKACB	NM_182948	2, 3, 4	Fusion	5'
PRKCA	NM_002737	4, 5, 6, 9, 15	Fusion	5'
PRKCB	NM_002738	1, 3, 7, 8, 9	Fusion	5'
PRKCD	NM_006254	9, 10, 11, 12, 15	Fusion	5'
PRKCD	NM_006254	18	Fusion	3'
PRKD1	NM_002742	2, 10, 11, 12, 13	Fusion	5'
PRKD2	NM_016457	10, 11, 12, 13	Fusion	5'
PRKD3	NM_005813	10, 11, 12, 13	Fusion	5'
RAD51B	NM_133509	8	Fusion	5'
RAD51B	NM_133509	3, 4, 5, 6, 7, 8, 9	Fusion	3'
RAF1	NM_002880	2*, 4, 5, 6, 7, 8, 9, 10, 11, 12	Fusion	5'
RAF1	NM_002880	4, 5, 6, 7, 8, 9	Fusion	3'
RELA	NM_021975	1, 2, 3, 4, 11	Fusion	5'
RET	NM_020630	2, 4, 6, 8, 9, 10, 11, mid-exon11, 12, 13, 14	Fusion	5'
RET	NM_020630	15, 16	Mutation	p.A883, p.M918
ROS1	NM_002944	2, 4, 7, 31, 32,33, 34, 35, 36, 37	Fusion	5'
ROS1	NM_002944	38	Mutation	p.G2032
RSPO2	NM_178565	1, 2*, 3*	Fusion	5'
RSPO3	NM_032784	2	Fusion	5'
SS18	NM_001007559	2, 3, 4, 5, 6, 10, 11	Fusion	5'
SS18	NM_001007559	4, 5, 6, 8, 9, 10	Fusion	3'
SS18L1	NM_198935	1, 2, 3, 8, 9, 10	Fusion	3'

STAT6	NM_001178078	1*, 2*, 3, 4, 5, 6, 7, 15, 16, 17, 18, 19, 20	Fusion	5'
TAF15	NM_139215	6, 7	Fusion	5'
TAF15	NM_139215	5, 6, 7, 9	Fusion	3'
TCF12	NM_207036	4, 5, 6	Fusion	3'
TERT	NM_198253	2, 3, 5, 7, 10, 11, 12	Fusion,Expression	5'
TERT	NM_198253	3, 9, 15	Fusion,Expression	3'
TFE3	NM_006521	2, 3, 4, 5, 6, 7, 8	Fusion	5'
TFE3	NM_006521	2, 3, 4, 5, 6	Fusion	3'
TFEB	NM_007162	1*, 2*, 3, mid- exon3, 4, mid-exon4, mid-exon 5, 6	Fusion	5'
TFEB	NM_007162	9, mid-exon 10	Fusion	3'
TFG	NM_006070	6	Fusion	5'
TFG	NM_006070	3, 4, 5, 6, 7, mid-exon8	Fusion	3'
THADA	NM_022065	24, 25, 26, 27, 28, 29, 30, 31, 36, 37	Fusion	3'
TMPRSS2	NM_001135099	1	Fusion	3'
TMPRSS2	NM_005656	1*, 2, 3, 4, 5, 6	Fusion	3'
USP6	NM_004505	1*, mid-exon1*,2*, 3	Fusion	5'
VGLL2	NM_182645	1, 2, 3, intron3, 4	Fusion	3'
WWTR1	NM_015472	3, 4	Fusion	5'
WWTR1	NM_015472	3, 4	Fusion	3'
YAP1	NM_001130145	1, mid-exon1, 2, 3, 4, 8, 9	Fusion	5'
YAP1	NM_001130145	1, 2, 3, 4, 5, 6, 7	Fusion	3'
YWHAE	NM_006761	5	Fusion	3'

*Indicates exons that are entirely untranslated region (UTR), or for which the UTR is targeted.
**The mutations listed are targeted by the assay design. Version 6.3 and earlier of Archer Analysis may not support RNA SNV/InDel variant calling at exon junctions depending on the sequence context (SNVs ≤5bp, Indels ≤30bp). RNA SNV/InDel mutation detection is not supported on the Ion Torrent Sequencing Platform
$^{\gamma}$ ALK-ATI currently requires review outside of Archer Analysis

Supplementary Figure S1

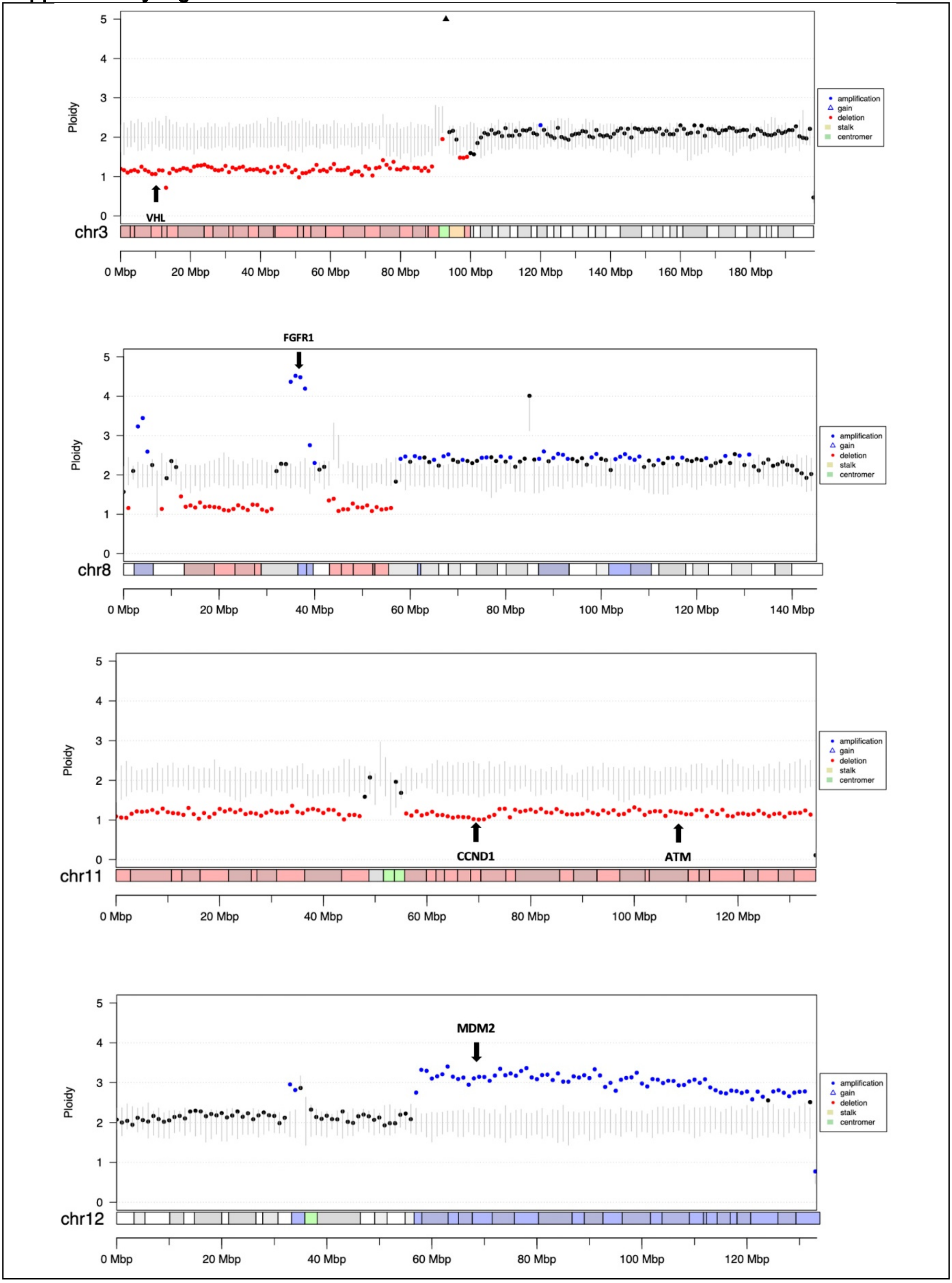

Supplementary Figure S1. Chromosome (Chr.) plots generated by CORIANDR for Chr. 3, 8, 11, 12 of a patient with sarcoma. In addition to the CNAs underlined in the Manuscript (Figure 2), ulcWGS uncovered partial losses in Chr. 3 (del(p)), Chr. 8 (del(p22p21.1), add(p11.23p11.22)x2, del(p11.1q11.23), Chr. 11 (-11) and Chr. 12 (add(q13.3q24.33). These alterations are compatible with low-level copy number (CN) gains and losses also reported by the VP panel: VHL (CN: 0,54), FGFR1 (CN: 2,28), CCND1 (CN: 0,59), ATM (CN: 0,55), MDM2 (CN: 1,45).

Supplementary figure S2

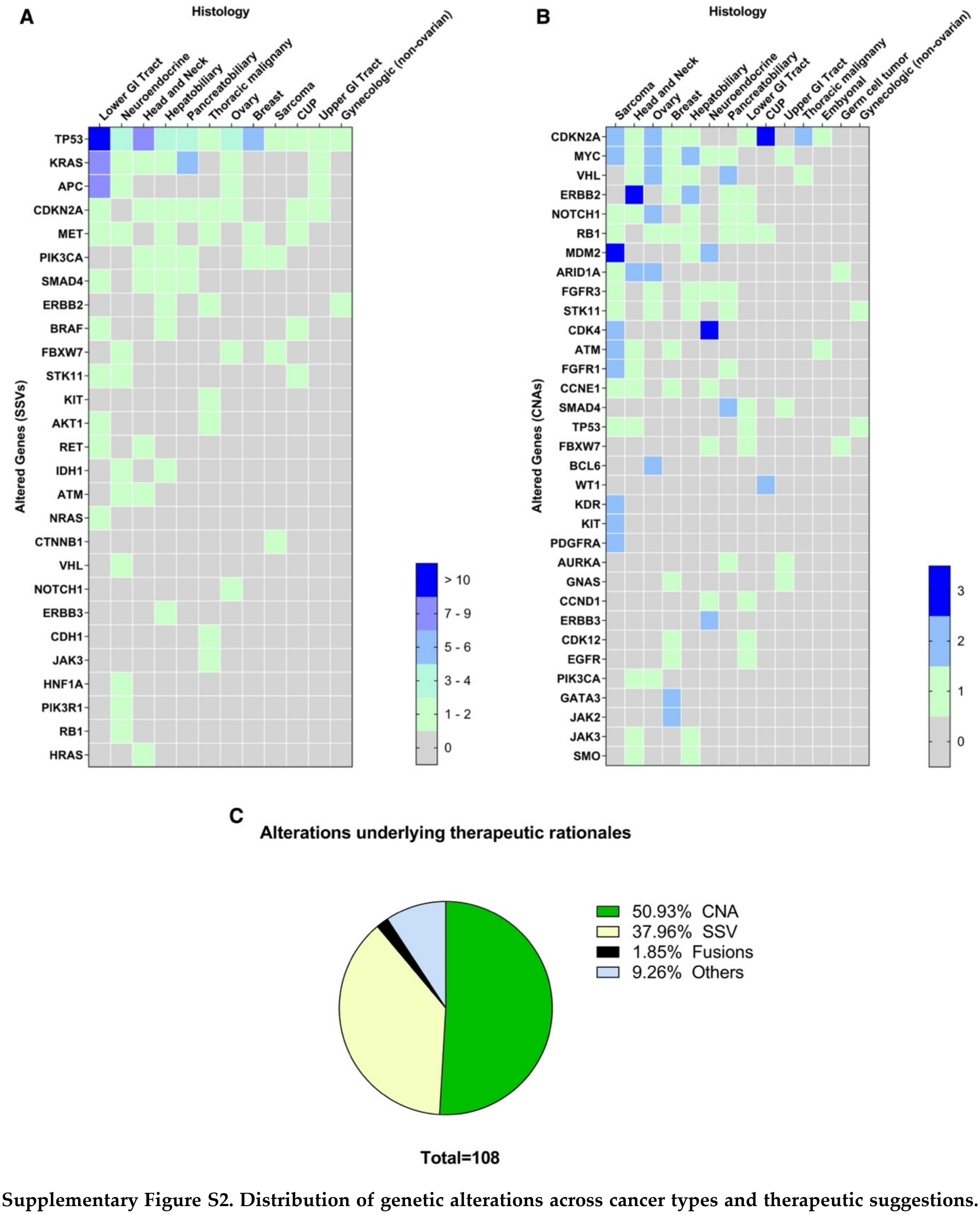

Supplementary Figure S2. Distribution of genetic alterations across cancer types and therapeutic suggestions. (A) Distribution of SSVs across different tumor groups. (B) Distribution of CNAs across different tumor groups. (C) Alterations determining therapeutic suggestions in the MTB cohort.

Supplementary Figure S3

A

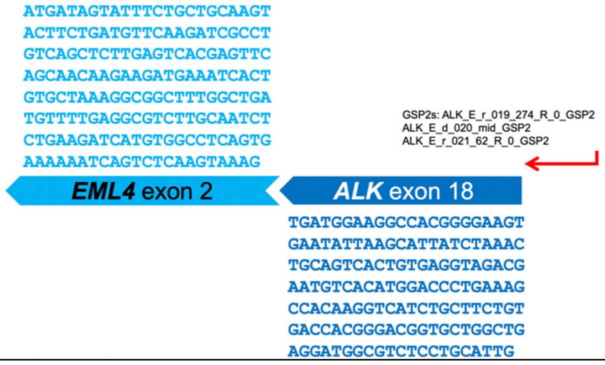

**Breakpoints**	chr2:42472827, chr2:29449940
**5'-Partner mRNA**	chr2:42472645→42472827 EML4(+) NM_019063.4, exon:2
**3'-Partner mRNA**	chr2:29449940→29449788 ALK(-) NM_004304.4, exon:18
**Reads (#/%)**	507 / 88.33%
**Start Sites**	71

B

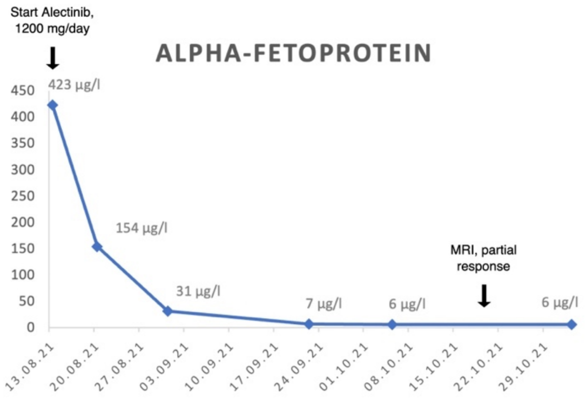

C

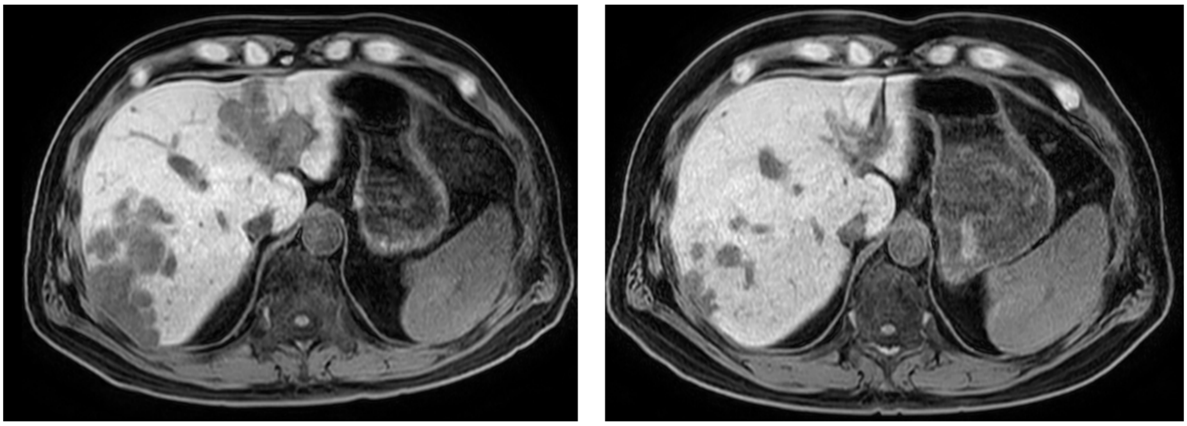

MRI liver scan (T1) before initiation of treatment with Alectinib

MRI liver scan (T1) 2 months after initiation of treatment with Alectinib

Supplementary Figure S3: A non-canonical *EML4-ALK* fusion in a patient with pancreatic cancer. We report the first case, to our knowledge, of a non-canonical *EML4-ALK* translocation that originated from breakpoints of *EML4* exon 2 and *ALK* exon 18 (Figure S2A) detected in a patient with metastatic pancreatic adenocarcinoma. Since another case of non-canonical *EML4-ALK* with *ALK* exon 18 has been reported to be refractory to crizotinib [10], a second generation ALK inhibitor (Alectinib) was started at the standard dose of 600 mg twice daily. The patient immediately showed clinical response, decrease of tumor marker values (Alpha-Fetoprotein) (Figure S2B) and partial remission in magnetic resonance imaging (MRI) (Figure S2C).

Supplementary Table S4

Patients with major clinical benefit following MTB recommended therapy

Diagnosis	Therapeutic rationale	MTB recommendation	EL ESCAT	EL DKTK	Label	PFS2	PFS1	PFSr	Outcome
CUP	*TMPRSS2-ERG* Fusion (changed diagnosis from CUP to prostate cancer)	Docetaxel + ADT	n/a	n/a	On	6	3.5	1.7	SD for 6 months
Acinic cell carcinoma	*ATM* del	Niraparib + Carboplatin	III-B [11]	m4	Off	12	n/a	n/a	SD for 12 months
CUP	*SEC31A-ALK* Fusion (changed diagnosis from CUP to CD20-negative *ALK*+ DLBCL)	Chemotherapy (CHOEP)	n/a	n/a	On	23.5	10.5	2.2	CR, ongoing at almost 2 year follow-up
CRC	*BRAF* V600E	Encorafenib + Binimetinib + Cetuximab	I-A [12]	m1a	Off	7.5	3	2.5	SD for 7.5 months
Sinonasal adenocarcinoma	*ARID1A* deletion	Pembrolizumab	III-A [13]	m2b	Off	12	n/a	n/a	SD for 12 months
Steroid cell tumor	*RET* Y791F	Cabozantinib	IV-A [14]	m3	Off	9.5	1	9.5	Decrease of Tumor marker and SD for 9.5 months
CUP	*BRAF* V600E	Encorafenib + Binimetinib	III-A [15]	m2a	Off	18.5	3	6.2	PR and SD for 18 months
TNBC	*EGFR* amplification	Cetuximab + Capecitabine	IV-A [16]	m1c [17]	Off	9.25	2	4.6	SD for 9.25 months
Sarcoma	*KDR, KIT, PDGFRA* Amplification	Pazopanib	IV-A [18]	m3	On	6.25	14	0.4	SD for 6.25 months
Pancreatic cancer	*EML4-ALK* Fusion	Alectinib monotherapy	III-B [19]	m4	Off	7.7	1	7.7	PR and SD for 7.7 months

Supplementary Table S5. Genetic alterations in *n*=104 patients referred to the MTB.

See separate xlsx-file

Supplementary References

1. Langmead B, Salzberg SL. Fast gapped-read alignment with Bowtie 2. Nature Methods. 2012 Apr;9(4):357–9.
2. Li H. A statistical framework for SNP calling, mutation discovery, association mapping and population genetical parameter estimation from sequencing data. Bioinformatics. 2011 Nov;27(21):2987–93.
3. Liao Y, Smyth GK, Shi W. FeatureCounts: An efficient general purpose program for assigning sequence reads to genomic features. Bioinformatics. 2014 Apr 1;30(7):923–30.
4. Quinlan AR. BEDTools: The Swiss-Army tool for genome feature analysis. Current Protocols in Bioinformatics. 2014;2014:11.12.1-11.12.34.
5. Benjaminit Y, Hochberg Y. Controlling the False Discovery Rate: a Practical and Powerful Approach to Multiple Testing. Vol. 57, J. R. Statist. Soc. B. 1995.
6. Cheung et al. Integration of cytogenetic landmarks into the draft sequence of the human genome. Nature. 2001;953–8.
7. An International System for Human Cytogenomic Nomenclature [Internet]. 2020. Available from: https://www.karger.com/Book/Home/279152
8. Papaemmanuil E, Gerstung M, Bullinger L, Gaidzik VI, Paschka P, Roberts ND, et al. Genomic Classification and Prognosis in Acute Myeloid Leukemia. New England Journal of Medicine. 2016 Jun 9;374(23):2209–21.
9. Bailey MH, Tokheim C, Porta-Pardo E, Sengupta S, Bertrand D, Weerasinghe A, et al. Comprehensive Characterization of Cancer Driver Genes and Mutations. Cell. 2018 Apr 5;173(2):371-385.e18.
10. Anai S, Takeshita M, Ando N, Ikematsu Y, Mishima S, Ishida K, et al. A case of lung adenocarcinoma resistant to crizotinib harboring a novel EML4-ALK variant, exon 6 of EML4 fused to exon 18 of ALK. Vol. 11, Journal of Thoracic Oncology. Lippincott Williams and Wilkins; 2016. p. e126–8.
11. Mateo J, Carreira S, Sandhu S, Miranda S, Mossop H, Perez-Lopez R, et al. DNA-Repair Defects and Olaparib in Metastatic Prostate Cancer. N Engl J Med. 2015 Oct 29;373(18):1697–708.
12. Kopetz S, Grothey A, Yaeger R, van Cutsem E, Desai J, Yoshino T, et al. Encorafenib, Binimetinib, and Cetuximab in BRAF V600E–Mutated Colorectal Cancer . New England Journal of Medicine. 2019 Oct 24;381(17):1632–43.
13. Okamura R, Kato S, Lee S, Jimenez RE, Sicklick JK, Kurzrock R. ARID1A alterations function as a biomarker for longer progression-free survival after anti-PD-1/PD-L1 immunotherapy. J Immunother Cancer. 2020;8(1).
14. Bentzien F, Zuzow M, Heald N, Gibson A, Shi Y, Goon L, et al. In vitro and in vivo activity of cabozantinib (XL184), an inhibitor of RET, MET, and VEGFR2, in a model of medullary thyroid cancer. Thyroid. 2013 Dec 1;23(12):1569–77.
15. Dummer R, Ascierto PA, Gogas HJ, Arance A, Mandala M, Liszkay G, et al. Encorafenib plus binimetinib versus vemurafenib or encorafenib in patients with BRAF -mutant melanoma (COLUMBUS): a multicentre, open-label, randomised phase 3 trial. The Lancet Oncology. 2018 May;19(5):603–15.
16. Zhu H, Wang C, Wang J, Chen D, Deng J, Deng J, et al. A subset of esophageal squamous cell carcinoma patient-derived xenografts respond to cetuximab, which is predicted by high EGFR expression and amplification. Journal of Thoracic Disease. 2018 Sep;10(9):5328–38.
17. Sabatier R, Lopez M, Guille A, Billon E, Carbuccia N, Garnier S, et al. High Response to Cetuximab in a Patient With *EGFR* -Amplified Heavily Pretreated Metastatic Triple-Negative Breast Cancer. JCO Precision Oncology. 2019 Dec;(3):1–8.

18. Kiyuna T, Murakami T, Tome Y, Igarashi K, Kawaguchi K, Miyake K, et al. Doxorubicin-resistant pleomorphic liposarcoma with PDGFRA gene amplification is targeted and regressed by pazopanib in a patient-derived orthotopic xenograft mouse model. Tissue & cell. 2018 Aug;53:30–6.
19. Camidge DR, Dziadziuszko R, Peters S, Mok T, Noe J, Nowicka M, et al. Updated Efficacy and Safety Data and Impact of the EML4-ALK Fusion Variant on the Efficacy of Alectinib in Untreated ALK-Positive Advanced Non–Small Cell Lung Cancer in the Global Phase III ALEX Study. Journal of Thoracic Oncology. 2019 Jul 1;14(7):1233–43.
